# A High Throughput Screening Assay System for the Identification of Small Molecule Inhibitors of *gsp*

**DOI:** 10.1371/journal.pone.0090766

**Published:** 2014-03-25

**Authors:** Nisan Bhattacharyya, Xin Hu, Catherine Z. Chen, Lesley A. Mathews Griner, Wei Zheng, James Inglese, Christopher P. Austin, Juan J. Marugan, Noel Southall, Susanne Neumann, John K. Northup, Marc Ferrer, Michael T. Collins

**Affiliations:** 1 Skeletal Clinical Studies Unit, Craniofacial and Skeletal Diseases Branch, National Institute of Dental and Craniofacial Research, National Institutes of Health, Bethesda, Maryland, United States of America; 2 Department of Preclinical Innovation, National Center for Advancing Translational Sciences, National Institutes of Health, Bethesda, Maryland, United States of America; 3 Clinical Endocrinology Branch, Laboratory of Endocrinology and Receptor Biology, National Institute of Diabetes and Digestive and Kidney Diseases, National Institutes of Health, Bethesda, Maryland, United States of America; 4 Laboratory of Membrane Biochemistry and Biophysics, National Institute on Alcohol Abuse and Alcoholism, National Institutes of Health, Bethesda, Maryland, United States of America; Albert-Ludwigs-University, Germany

## Abstract

Mis-sense mutations in the α-subunit of the G-protein, G_s_α, cause fibrous dysplasia of bone/McCune-Albright syndrome. The biochemical outcome of these mutations is constitutively active G_s_α and increased levels of cAMP. The aim of this study was to develop an assay system that would allow the identification of small molecule inhibitors specific for the mutant G_s_α protein, the so-called *gsp* oncogene. Commercially available Chinese hamster ovary cells were stably transfected with either wild-type (WT) or mutant G_s_α proteins (R201C and R201H). Stable cell lines with equivalent transfected G_s_α protein expression that had relatively lower (WT) or higher (R201C and R201H) cAMP levels were generated. These cell lines were used to develop a fluorescence resonance energy transfer (FRET)–based cAMP assay in 1536-well microplate format for high throughput screening of small molecule libraries. A small molecule library of 343,768 compounds was screened to identify modulators of *gsp* activity. A total of 1,356 compounds with inhibitory activity were initially identified and reconfirmed when tested in concentration dose responses. Six hundred eighty-six molecules were selected for further analysis after removing cytotoxic compounds and those that were active in forskolin-induced WT cells. These molecules were grouped by potency, efficacy, and structural similarities to yield 22 clusters with more than 5 of structurally similar members and 144 singleton molecules. Seven chemotypes of the major clusters were identified for further testing and analyses.

## Introduction

McCune-Albright syndrome (MAS) is a rare disease that arises as the result of mutations in the alpha subunit of the G_s_ protein (G_s_α) encoded by *GNAS* [1], [2]. G_s_α is a protein central to G-protein coupled receptor (GPCRs) signal transduction, and as such is involved in some aspect of nearly every physiologic pathway and organ system. The G_s_α mutations (sometimes referred to as the *gsp* oncogene) arise postzygotically in MAS. Therefore patients with MAS have the mutation in a mosaic pattern with varying degrees of tissue involvement ranging from a single site within a single tissue with almost no disability to widespread distribution that may be lethal [3], [4]. The prevailing understanding is that if these mutations were germline they would be lethal, and that the mutation “survives” through somatic mosaicism [5]. To date, this concept is supported by the absence of any cases resulting from vertical transmission and discordance in disease among monozygotic twins. Additional clinical significance of these mutations is the fact they are also found in sporadic hyperfunctioning endocrine tumors, pancreatic tumors, and various other cancers [6]–[8].

Greater than 90% of the mutations in G_s_α in MAS occur at the R201 position and are relatively equally divided between R201H and R201C [3]. The R201 residue resides in the GTPase pocket and is necessary for termination of GPCR signaling [9]. The H and C mutations lead to loss or impairment of the intrinsic GTPase activity and protracted signaling [10]. Thus, these activating mutations lead to ligand-independent increases in cAMP that result in altered downstream signaling and gene expression in affected tissues. The tissue phenotype varies by the function of the given cell and is the result of downstream activation in that cell type. For example, melanocytes overproduce melanin in a melanocyte stimulating hormone-independent fashion resulting in café-au-lait skin spots [11]. Likewise, pituitary somatotrophs overproduce growth hormone in a growth hormone releasing hormone-independent fashion resulting in gigantism/acromegaly [12]. Skeletal stem cells in bone marrow behave as if they are under constant parathyroid hormone stimulation and fail to differentiate into mature osteoblasts and osteocytes and instead proliferate as immature osteogenic cells leading to the marrow fibrosis and fibrous dysplasia of bone (FD), which are histopathologically similar to brown tumors seen in hyperparathyroidism [13].

Prominent clinical features of MAS are café-au-lait spots, fibrous dysplasia of bone, precocious puberty, growth hormone excess, hyperthyroidism, cortisol excess (Cushing syndrome), hepatic dysfunction, and an ill-defined neuropsychiatric syndrome [14]. In its most severe forms, when the mutation arises very early in embryonic development and nearly all tissues are involved, MAS can be lethal. Tissue/organ-specific treatments exist for some aspects of the disease with varying degrees of efficacy. There are no directed treatments available for FD.

The identification of molecules that specifically target *gsp* mutations would be beneficial, both as probes for the study of the altered signaling as well as the basis for the development of drugs to treat FD/MAS and other disorders arising from *gsp* mutations. For this purpose, we created several cell lines that overexpress either the wild-type or mutated G_s_α protein. One cell line from each set, wild type, R201C, and R201H (WT9, C6 and H2 cells) was selected for further study. cAMP levels were measured in either an ELISA or fluorescence resonance energy transfer (FRET)-based assay and optimized in 96- and 1536-well formats. Various established adenylyl cyclase inhibitors and activators were used to confirm that cAMP levels could be inhibited or stimulated in these cell lines. First, a small molecule library consisting of 1280 pharmacologically active compounds (LOPAC, Sigma-Aldrich, St. Louis, MO) was tested with the R201C mutant (C6) cell line to assess the robustness of the assay before proceeding to a full library screening. Next, the Molecular Libraries Small Molecule Repository (MLSMR) library of 343,768 compounds was screened for inhibition of cAMP levels. 1356 compounds were selected as active based on their efficacy and retested as concentration dose responses to determine both potency and efficacy. These 1356 compounds underwent additional testing for cytotoxicity effects, and activity in a forskolin-stimulated cAMP assay in WT cells. Six hundred eighty-six compounds were identified that specifically inhibited the cAMP levels in the mutated G_s_α cell line, but had no effect on forskolin-stimulated WT cells. These molecules were analyzed based on structure similarities and 22 clusters and 144 singletons were identified, including 7 major clusters.

## Materials and Methods

### Establishment of cell lines expressing wild type (WT) and mutated G_s_α

The coding regions from wild-type and mutated G_s_α were subcloned into a pcDNA3.1/myc-His expression vector (Invitrogen, Carlsbad, CA), as previously described, and the sequence confirmed (Figure S1) [15]. Cell sensor CRE-bla CHO-K1 cells (Invitrogen, Carlsbad, CA) were grown in Dulbecco's modified Eagle's medium (DMEM; Life Technologies, Carlsbad, CA) containing 10% fetal bovine serum (HyClone, Logan, UT), Penicillin (100 U/ml)-Streptomycin (100 µg/ml), L-glutamine, 25 mM HEPES, pH 7.3, 1 mM non-essential amino acids (NEAA), and 1 mM sodium pyruvate (Invitrogen, Carlsbad, CA). Cells were seeded (1.5×10^6^/100 mm plate) and grown to 80% confluence prior to transfection. Cells were transfected in serum-free condition by using 2 or 10 µg each of expression plasmids and Lipofectamine 2000 (Invitrogen, Carlsbad, CA) and incubating at 37°C for 3–5 hours. Transfection was monitored by microscopy and the presence of green fluorescence on a separate plate that was transfected with YFP-N1 (Clontech, Mountain View, CA). After 24 hours of transfection, the medium was changed and cells were grown in full culture medium containing 1000 µg/ml gentamycin (Invitrogen, Carlsbad, CA) for selection purposes. Cells were grown in selection medium for 2–3 weeks until cell colonies formed. Colonies were selected using Cloning Cylinders (Millipore, Bedford, MA) and propagated separately. Several clones were propagated for use in further screening steps. Four separate clones from individual stable clones were used for cAMP measurement using a cAMP ELISA kit (Tropix cAMP-Screen direct cyclic AMP ELISA system, Applied Biosystems, Foster City, CA).

### Confirmation of equivalent expression of transfected genes

Cell extracts were prepared from each cell line using whole cell extract buffer (10% glycerol, 250 mM NaCl, 10 mM HEPES pH 7.5, 1 mM EDTA, 0.1% NP-40, 1 mM DTT and 1× protease inhibitor cocktail) (CalBiochem EMD Biosciences, La Jolla, CA). Protein concentrations were measured using BCA Protein Assay Kit (Thermo Scientific, Rockford, IL). Equal amounts of protein extracts were loaded on 4–20% polyacrylamide gel (Bio-Rad, Hercules, CA) and were then transferred onto nitrocellulose paper for immunodetection purposes using Anti-c-Myc antibody (Invitrogen, CA). Loading for each lane was normalized by using mouse anti-human β-actin antibody (Sigma, St. Louis, MO) (Figure S2).

### Demonstration of differences in cAMP levels

cAMP levels were determined in stable clones by ELISA (Tropix cAMP-Screen direct cyclic AMP ELISA system, Applied Biosystems, Foster City, CA). Stable cell lines (1×10^4^ cells/96-well) were grown in full medium overnight and incubated for 30 minutes in fresh serum-free medium containing 10–50 µM of 3-isobutyl-1-methylxanthine (IBMX) (Sigma, St. Louis, MO), a known phosphodiesterase inhibitor. Cell and medium were collected and the amount of cAMP was measured following the manufacturer's instructions. One cell line from each set (WT9 cells expressing the wild-type G_s_α, C6 for cells expressing the R201C G_s_α and H2 for cells expressing the R201H G_s_α) was selected for further studies (Figure S3).

Results from light microscopy also showed that the C6 and H2 cell lines demonstrate a more fibroblastic/stellate appearance relative to WT9 cells, consistent with morphologic changes typically induced by higher cAMP levels, as has been previously demonstrated (Figure S4) [16].

C6 cells carrying the R201C mutation exhibited very high basal cAMP levels compared to WT9 cell line, and were therefore used for the subsequent experiments. The assay was optimized for cell density and phosphodiesterase inhibitor. Cell densities of 1000–3000 cells/well did not show significant differences in basal cAMP level (Figure S5A). There were significant differences in basal cAMP levels with the addition of Ro-20-1724 (Figure S5B), though there were no major differences in the cAMP levels from 30–120 minutes (data not shown). Therefore, a 2000 cells/well density, a 30 min incubation period, and the inclusion of Ro-20-1724 were chosen as optimized conditions for the assay.

### Demonstration of system inhibition and stimulation

To demonstrate that the system would be able to detect molecules that would inhibit and stimulate cAMP, adenylyl cyclase inhibitors and stimulators were tested. Cells were grown in either 96-well (5–10×10^3^ cells/well) overnight in full medium. The medium was then changed to core DMEM containing 50 µM of the phosphodiesterase inhibitor 4-(3-Butoxy-4-methoxybenzoyl)-2-imidazolidine (Ro-20-1724) (Sigma, St. Louis, MO) for 1–2 hours. The medium was then removed from each well and treatments were started using the same medium containing Ro-20-1724 for 30 minutes or other specified times at 37°C. cAMP levels were measured in the cells after treatment with four different adenylyl cyclase inhibitors [2′,5′-dideoxyadenosine (ddA)], (E)-2-(1H-Benzo[d]imidazol-2-ylthio)-N′-(5-bromo-2-hydroxybenzylidene) propanehydrazide (KH7), MDL-12,330A (MDL) and SQ 22,536 (SQ)], and the adenylyl cyclase activator, forskolin (compounds were purchased from Sigma, St. Louis, MO). Different concentrations of inhibitors were tested on C6 (expressing R201C) and H2 (expressing R201H) cells for 5, 15 and 30 minutes in 96-well formats. All the treatments were performed at 37°C for 5–60 minutes. A fluorescence resonance energy transfer (FRET)-based cAMP assay kit (Cisbio, Bedford, MA) was used to measure cAMP (Figure S6A–D). 1 µM ddA or SQ could inhibit the basal levels of cAMP by 60–75% after 5 minutes of incubation. The degree of inhibition was variable in the cells treated with KH7 or MDL. The less robust and more variable effect of KH7 and MDL may be due to the fact that KH7 may be more of an inhibitor of soluble rather than membrane-bound adenylyl cyclase and that MDL has recently been shown to have activity on pathways unrelated to adenylyl cyclase [17]. There was an approximately 10-fold increase in basal cAMP levels in C6 cells after forskolin treatment for 30 minutes (Figure S6E). These results were similar in the H2 cells (data not shown). These data indicated that the cAMP levels in cells expressing mutant G_s_α could be inhibited and stimulated, and therefore appropriate for HTS screening.

### High throughput screen (HTS) assay

Cells were plated (2,000 cells/well) in 6 µl of complete medium (DMEM with 10% fetal bovine serum and 1% penicillin/streptomycin, 0.5 mg/ml G418) 1536-well plates and grown overnight at 37°C 5% CO_2_ and 95% humidity. Certain cells, both *in vivo* (proximal renal tubule cells) and cell lines have the intrinsic capacity to export cAMP. This transport is typically probenecid-responsive, in which probenecid inhibits cAMP transport. Export of cAMP to the media is a property of the CHO cell line used in this assay (Figure S7). Therefore, to maximize the ability to detect inhibitor-induced changes in cAMP accumulation in the conditioned medium that occurred during the overnight incubation period, a wash protocol was included (Table 1). Cells were seeded (2,000 cells/well) in 6 µl DMEM. Following the overnight incubation, the growth media was aspirated leaving 1 µl residual volume. The wells were washed with 5 µl DPBS containing 1 mM CaCl_2_, 0.5 mM MgCl_2_, 0.05% BSA, 0.005% Tween 20, then aspirated leaving 2 µl residual volume. To each well 23 nl/well of test compounds in DMSO were dispensed via pin transfer before 1 µl medium containing 300 µM Ro 20-1724, a known phosphodiesterase inhibitor, was added. The cells were incubated with compounds for 30 minutes at 37°C. HRTF assays were performed using the homogenous time-resolved fluorescence (HTRF) HiRange cAMP detection kit (Cisbio, Bedford, MA) by adding 1.0 µl/well cAMP-conjugated d2 and 1.0 µl/well europium cryptate conjugated anti-cAMP antibody in lysis buffer according to the manufacturer's instructions. Plates were incubated at room temperature for 30 minutes and FRET signals (665 and 615 nM) were read using an EnVision plate reader (PerkinElmer, Waltham, MA). HTRF signal was calculated as the ratio of signal from the 665 nm (acceptor) and 615 nm (donor) channels and multiplied by 10,000. % activity was calculated by normalizing each the HTRF signal from each sample well to the mean HTRF signal from the DMSO only control wells.

**Table 1:** Assay Protocol.

Step	Parameter	Value	Description*
1	Reagent	6 µl	2000 cells/well in 6 µl/well DMEM with 10% FBS, 1× Penicillin/Streptomycin, 0.5 mg/ml G418
2	Incubation	16–24 hr	37°C, 5% CO_2_, 95% humidity
3	Aspirate	−5 µl	Leave 1 µl residual
4	Reagent	5 µl	DPBS, 1 mM CaCl_2_, 0.5 mM MgCl*_2_*, 0.05% BSA, 0.005% Tween 20
5	Aspirate	−4 µl	Leave 2 µl residual
6	Compound	23 nl	Columns 1–4 controls, columns 5–48 compounds
7	Reagent	1 µl	300 µM Ro-20174 in DMEM (no phenol) 10% FBS
8	Incubation	30 min	37°C, 5% CO_2_, 95% humidity
9	Reagent	1 µl	HTRF kit: cAMP-d2 in lysis buffer
10	Reagent	1 µl	HTRF kit: anti-cAMP-K in lysis buffer
11	Incubation	30 min	Room temperature
12	Detection		EnVision plate reader; HTRF mode (excitation at 320 nM, and emission at 615 nm and 665 nm)

*See Materials and Methods for more details, definitions, and non-standard abbreviations. Ro-20174 = 4-(3-Butoxy-4-methoxybenzoyl)-2-imidazolidine, HTRF = homogeneous time resolved fluorescence.

### Screening of Library of Pharmacologically Active Compounds (LOPAC)

To test feasibility of proceeding to a full screening assay, the LOPAC library (1280 compounds, Sigma-Aldrich, St. Louis) was screened using a quantitative qHTS assay format as described previously (Table S1) [18]. Prior to that screen, a 1536-well DMSO test plate (solvent control), was tested for signal-to-basal (S/B) ratio and Z′ factor calculations (Figure S8). The S/B and Z-factor were calculated from 16 control wells each with CHO-WT and CHO-C6 HTRF values. For the LOPAC screen, each compound was titrated in 5 concentrations. The final concentrations of the compounds assayed ranged from 92 nM to 57.5 µM. The mean S/B was 1.6 between DMSO and forskolin treatments, and 1.6 between DMSO and ddA treatments. The mean Z-factor was 0.24 between DMSO and forskolin treatments and 0.16 between DMSO and ddA treatments. Considering the fact that the assay requires two wash steps to remove secreted cAMP in the conditioned media, and that the assay is tuned to detect both increases and decreases in cAMP levels, the S/B and Z′ factors, while low, indicate that the assay is capable of detecting both an increase and decrease in cAMP levels. The screen resulted in the identification of several inhibitors and activators, and indicated that this assay was suitable for HTS (Tables S2, S3, S4A&B, Figures S8, S9A,B,&C).

### Curve response class classification from dose response HTS

Curve response classifications (CRCs) are the measure that includes potency, efficacy and reliability of the data, and estimates an IC_50_ value directly from the primary screen [18]. To determine CRCs the plate raw data were loaded into the NCATS quantitative high-throughput screening (qHTS) database and normalized to the DMSO and forskolin control wells. The data were then used to fit 4-parameter dose-response curves, a custom grid-based algorithm, to generate curve response class (CRCs) values for each compound [19]. The resultant curves were then classified using a heuristic curve classification scheme, allowing for the distinction of high quality curves (class 1.1) from lower (2.1, 1.2, 2.2) to poor quality ones (3, 4). Briefly, a curve (and hence a compound) was classified as 1.1 if it exhibited well defined upper and lower asymptotes, with a good fit to the observed data points (R^2^> = 0.9) and an efficacy greater than 80%. A class 2.1 curve was similar to a 1.1 curve, but exhibited only one well-defined asymptote. A curve that exhibited poorer efficacy (between 30% and 80%) was classified as a 1.2 or a 2.2 if it had two asymptotes or one asymptote, respectively. A class 3 curve was one that was poorly fit or only exhibited activity at the highest concentration, thus representing inconclusive activity, and a class 4 was assigned to those cases where there was no dose response, and considered inactive.

### Hit selection criteria

The following criteria were applied for hit selection from the primary screen: 1) hits in robust curve classes 1.1, 1.2, and 2.1 and active compounds in other curve classes (1.3/1.4/2.3/2.4/3) with maximum response (efficacy) >60% were considered active; 2) these hits were filtered for donor interference, and those compounds demonstrating donor interference were eliminated; 3) compounds were further filtered by reactive and promiscuous functional groups, as previously described [20]; 4) to group hits by structural similarity, clustering was performed using Leadscope Hosted Client (Leadscope Inc., Columbus, OH).

### Molecular Libraries Small Molecule Repository library screening

For the initial screen of the 343,768 compound MLSMR library, C6 cells, following HTRF cAMP assay protocol were screened at a single dose of compounds (38 µM). The screening assay was conducted according to the protocol outlined above (*High throughput screen (HTS) assay*, Table 1). A total of 1,375,072 wells were screened. The signal cut-off was set at >30% change in HTRF signal from basal activity. The mean S/B was calculated to be 1.66±0.30 and Z′-factor was 0.27±0.23.

### Forskolin-induced cAMP Assay in WT9 Cells

The WT9 clonal cell was selected based on its low basal level of cAMP, and robust stimulation with forskolin to induce detectable levels of cAMP using the HTRF assay. The cells were grown in DMEM, 10% FBS, 1% Pen/Strep, 0.5 mg/ml G418. The day before screening, 2000 cells/well in 3 µl in DMEM 10% FBS, 1% Pen/Strep, 0.5 mg/ml G418 were seeded in Greiner One high base solid bottom white tissue culture treated plates using a small cassette and a Multidrop (from Thermo Fisher). The plates were allowed to incubate 16–24 hr at 37°C, 5% CO2, 95% humidity covered with low evaporation stainless steel lids from Kalypsys. Prior to compound addition, 1 µl of 500 µM (final 100 µM) of the PDE inhibitor Ro-20174 solution in complete DMEM was dispensed to prevent degradation of cAMP. 23 nl of compound dose response solutions in DMSO were then dispensed using a Kalypsys pintool (diluted into 5 µL resulting in a 1∶217 dilution of compound). The control compound included the cAMP stimulator forskolin used at 4.6 µM final. Next, 1 µl of a 200 nM (final 40 nM) of forskolin solution in complete DMEM was added to induce an EC_80_ cAMP response. The plates were incubated for 30 minutes at 37°C, 5% CO2, 95% humidity using the same stainless steel lids. Finally, the high range cAMP HTRF kit (CisBio, Bedford, MA) was used to detect the levels of cAMP. A total 1 µl/well of the HTRF reagent cAMP-d2 in lysis buffer and 1 µl/well HTRF reagent anti-cAMP antibody-K in lysis buffer were dispensed at the same time using the FRD. The plates were incubated for 30 minutes at room temperature and then FRET signal was measured with an EnVision plate reader using an HTRF protocol (Excitation at 320 nM and Emission at 665 nM for the cAMP-d2 and 615 nM for the anti-cAMP antibody-K). The ratio of 615/615 nM is calculated to normalize for any effects in the donor only channel (Figure S10).

### HTS Viability Assays

For each cell line tested, a total of 500 cells per well in 5 µL of media was dispensed using a Multidrop Combi dispenser (Thermo Fisher Scientific Inc., Waltham, MA) and a small cassette into barcoded 1536 solid bottom white Greiner One tissue culture treated plates (catalog # 789173-F). The plates were then covered with stainless steel cell culture Kalypsys lids and incubated at 37°C with 5% CO_2_ under 95% humidity to allow the cells to adhere. Standard DMEM -1640 supplemented with 10% FBS, 1× penicillin/streptomycin/amphotericin, 2 mM glutamine and 0.5 mg/mL G418 was used (Gibco). For the generation of the standard 11 point dose response curves the library compounds and control compound forskolin (43 µM final) was added by the pintool addition (Kalypsys) of 23 nL solubilized in DMSO. The cells were incubated for 48 hours and then 3 µL of CellTiter Glo luminescent cell viability assay reagent (Promega, Madison, WI) was added using a Bioraptor Flying Reagent Dispenser (Aurora Discovery-BD). The plates were then incubated for 15 minutes at room temperature. The signal was captured using a 10 second exposure with a ViewLux (Perkin Elmer) contacting a luminescent filter. Relative luminescence units (RLU) for each well were normalized to the median RLUs from the DMSO control wells as 100% viability.

## Results and Discussion

The development of an assay to detect molecules that have activity at the mutated G_s_α that is responsible for FD/MAS, nonsyndromic hyperfunctioning endocrine tumors, pancreatic tumors, and various other cancers may be an important step towards developing drugs to treat these conditions. Currently there are no drugs that target *gsp*. Benign conditions such as FD, in which the *gsp* oncogene is the sole driver mutation and for which there are no effective treatments, and pre-malignant and malignant pancreatic neoplasms, which are at least in part driven by the *gsp* oncogene, will certainly benefit from drugs that can effectively and specifically target *gsp*. In this study, we describe the development of the assay used to identify compounds with activity at *gsp* and show the molecules identified. These molecules may represent a start towards the development of such drugs to treat these conditions.

**Figure 1:**
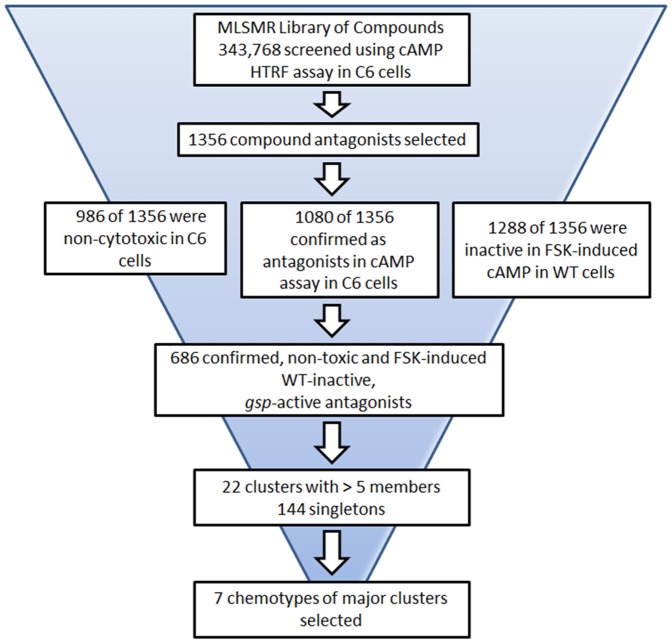
Compound Identification Flow Chart. Depicted is a flow chart of the assays, filtering, and analyses that were performed to ultimately identify the 7 chemotypes of clusters of molecules that have been selected for further study.

A flow chart outlining the results of the screen of the 343,768 compound MLSMR library are shown in Figure 1. The library was tested at a single dose compound dose of 38 µM. Primary actives from the HTS were selected based on % inhibition cut-off of >30%. These hits were then further filtered by removing those compounds with high or low signal in the donor channel, as well as for the presence of reactive and promiscuous functional groups. After these filters were applied, a total of 1356 inhibitory compounds were selected and re-tested in a seven concentrations dose response in the same cAMP as used for the primary HTS. The distribution by classification of CRCs from the confirmation screen is shown in Table 2. CRCs classify compound by both potency and efficacy. Compounds with CRC 1.1 are both potent and efficacious (>80% inhibition), and those with CRC 1.2 are less potent but efficacious. Sixteen compounds fit curve class 1.1 (0.005% of the library), and 175 fit curve class 1.2 (0.051% of the library). These compounds were counter-screened using WT9 cells that were sub optimally stimulated with forskolin, as well as for cytotoxic effects in the C6 cells. Results were analyzed by the CRC method as described in Materials and Methods. 1080 (80%) compounds were re-tested as active in the C6 cAMP assay; 1288 (95%) were unable to inhibit cAMP levels in the forskolin-stimulated WT cAMP assay, suggesting that these inhibitors act upstream of adenylyl cyclase, possibly *gsp*. 986 (73%) compounds tested non-cytotoxic. The combined cAMP, forskolin-stimulated WT and cytotoxicity screens yielded a total of 686 compounds that were selected for further analyses (Figure 1).

**Table 2:** Summary of Confirmation Hits.

Cherry Picks	Distribution	Curve Classification									
		1.1	1.2	1.3	1.4	2.1	2.2	2.3	2.4	3	4
1356	Compound Number	16	175	1	52	176	445	2	163	48	278
	%	1.18	12.91	0.07	3.83	12.98	32.82	0.15	12.02	3.54	20.5

**Figure 2:**
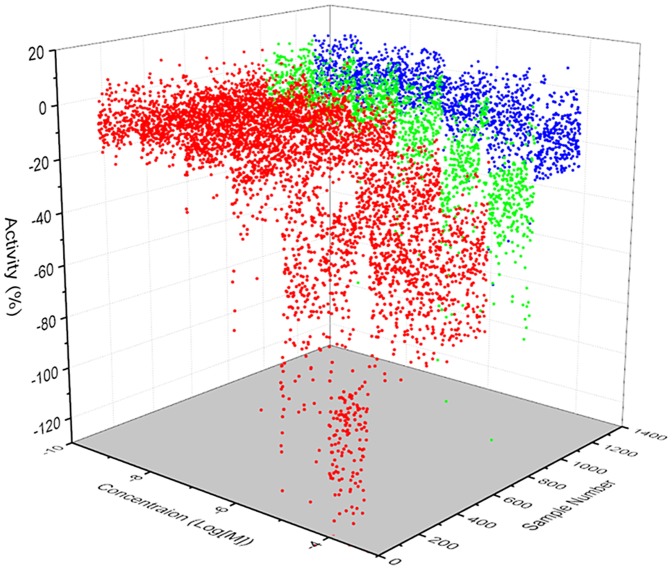
Confirmation Assay Molecules. A 3-axis plot of the 1356 compounds identified in the confirmation assay is shown. Compounds are sorted by curve class. Red: active compounds in curve class 1 and 2. Green: weakly active compounds in curve class 3. Blue: inactive compounds in curve class 4.

**Figure 3:**
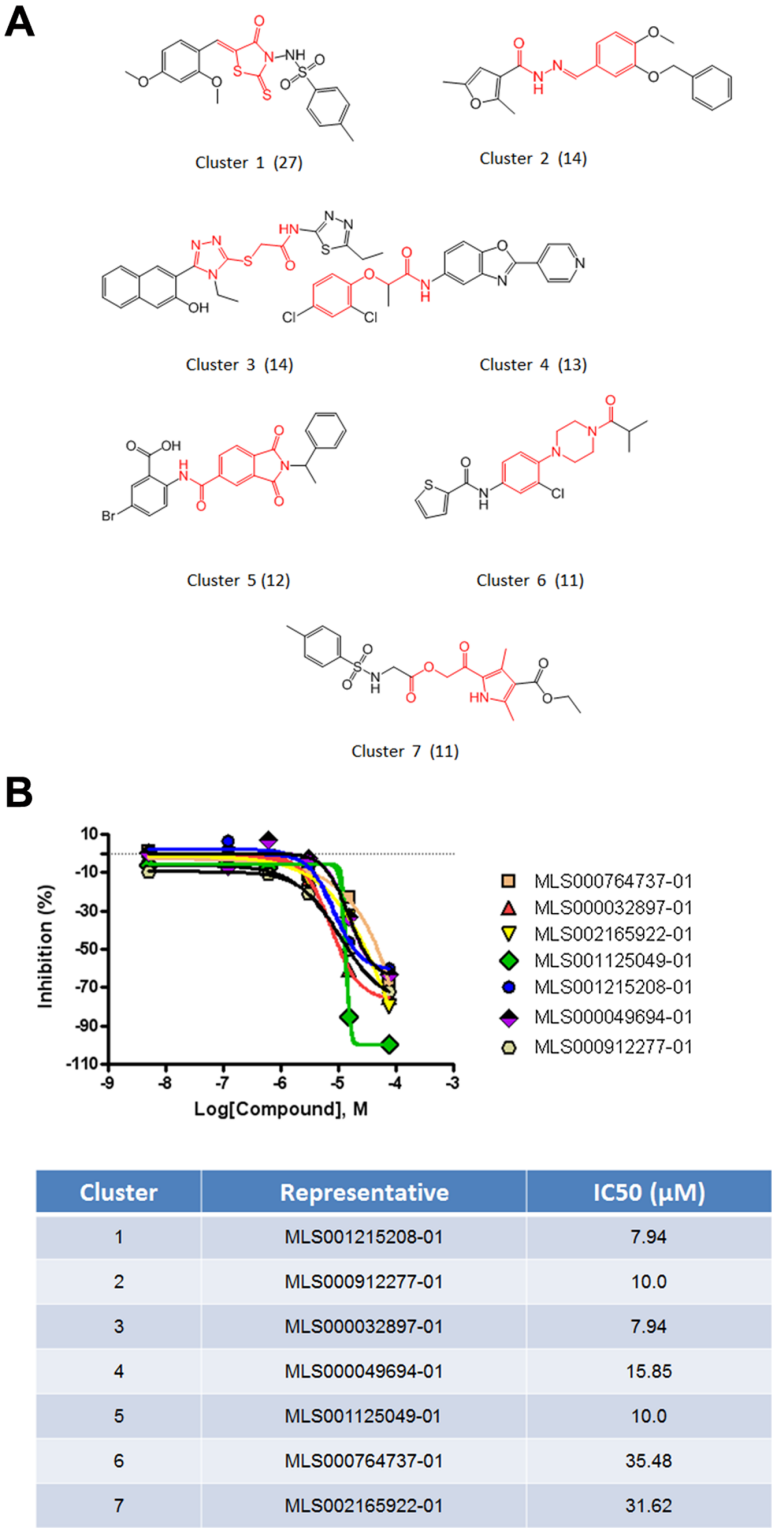
Clustering Analysis. Active compounds were clustered based on structural similarity to identify common chemotypes using LeadScope (Leadscope Hosted Client, Leadscope Inc., Columbus, OH). The results show a diversity of structural clusters, with 22 distinct clusters with more than 5 members. (A) Representative compounds from each of the most prominent 7 clusters are shown. (see Table S5, Cluster Analysis Compounds with Link for a complete list of the 102 molecules in the 7 clusters, their structures, IC_50_, and active link to the complete PubChem description). Their common structural scaffolds are highlighted in red. These scaffolds are highly polar, including thiazole, triazole, and hydrozide-based derivatives. Another common structural feature is that these small molecules share a linear molecular shape, which suggests that they might compete with GTP at the active site of the G protein. (B) Inhibition-concentration curves for 7 selected compounds, one from each cluster, together with the IC_50_ for each compound are shown.

qHTS profiling for these 686 selected compounds were performed using a three axis plot (Figure 2). The selected hits were clustered together by their structure-activity relationships (SAR) and by major structural similarities. There were 22 clusters with >5 members and 144 singletons identified. The seven clusters with the common structural scaffolds highlighted are shown in Figure 3A. A common feature of the molecular scaffold of the clusters was the fact they are highly polar, including thiazole, triazole, and hydrozide-based derivatives. An additional common structural feature is that these small molecules share a linear molecular shape. This suggests the possibility they could compete with GTP at the active site of the G protein.

Inhibition-concentration curves for representative molecules from each of the 7 major clusters are shown in Figure 3B. A complete list of the 102 compounds in this group of 7 clusters is found in Table S5 (Cluster Analysis with Link). Table S5 includes the molecule identifier, structure, IC50, and active link to a full description of the molecule in PubChem. A complete description of the screening assay was deposited into PubChem under PubChem bioassay identifier (AID): 624288 (http://pubchem.ncbi.nlm.nih.gov/assay/assay.cgi?aid=624288). Via this link one can access, among others, details on assay protocols, details on all 343,768 compounds screened in this assay grouped by active, inactive, and inconclusive compounds. One can also cross-reference other assays in which screened molecules have shown activity.

Additional testing with this group of molecules to demonstrate biological relevance and molecular specificity is needed. A potential system is the rat pituitary cell line, GH3. Similar to what is seen in patients with MAS, this is a somatolactotroph cell line that secretes both growth hormone and prolactin. Furthermore, it has been shown that when GH3 cells were transfected with an activated G_s_α (Q227L) cAMP levels and growth hormone and prolactin secretion were increased [21], [22]. Therefore, this cell line may represent an appropriate system for testing biological/clinical relevance.

A recent publication provides support that the mutant GTPase activity in G_s_α in fact may be able to be targeted by small molecules. Ostrem et al. were able to specifically target the G12C K-Ras mutant, which resides within the GTPase domain of K-Ras, a system strikingly similar to what is seen in MAS [23]. However, none of the molecules identified in our screen bear significant homology to those identified by Ostrem et al. This may owe to the fact that there are significant differences between size and shape of the Ras and G_s_α GTPase pockets. Nonetheless, this recent publication supports the feasibility of identifying *gsp* inhibitors.

In summary, an assay system for the identification of molecules with specific activity at the *gsp* mutation has been developed and identified a group of molecules available for further testing. Molecules identified in this screening may lead to both tools for the study of the GPCR/G_s_α/cAMP pathway as well as molecules from which drugs to treat diseases caused by *gsp* mutations can be developed.

## Supporting Information

Figure S1 **Wild Type and Mutant Clone Sequencing.** Wild type and mutant G_s_α clone sequencing. Recombinant plasmids carrying the WT (Arg201), Cys (R201C) and His (R201H) G_s_α were sequenced using an internal oligonucleotide to confirm the mutated area. Sequences from the pertinent area are shown.
(TIF)

Figure S2 **Stable Clones Express Equal Levels of Transfected G_s_α.** Equal Levels of Transfected G_s_α Expression. Mutant and WT transfected G_s_α was tagged with c-Myc to assess transfection efficiency. Cell lines were chosen that demonstrated equal amounts of c-Myc expression, reflecting equal transfected G_s_α expression. Cellular c-Myc, a molecular weight indicator and β-actin (loading control) are also labeled.
(TIF)

Figure S3 **cAMP Levels in Individual Wild Type and Mutant Clones.** Wild Type and Mutant Cell Line Performance. cAMP levels from individual stable clones expressing the YFP-N1 (control cells; Y, open bars), WT G_s_α (W, light grey bars), R201C G_s_α (C; dark grey bars) and R201H G_s_α (H, black bars) were measured using a cAMP ELISA assay. Assays were performed in triplicate and repeated at least three times.
(TIF)

Figure S4 **Cellular Morphology of G_s_α Clones in CRE-bla-CHO Cells.** Cellular Morphology in Response to Increased cAMP. Increased levels of cAMP were associated with a more fibroblastic appearance in transfected cells. This is an established phenomenon that results from increases in cAMP and is especially apparent in the C6 cell line. (see ref. 16).
(TIF)

Figure S5 **Cell Density and PDE Inhibitor Optimization.** The effect of cell density (A) and the phosphodiesterase inhibitor Ro-20-1724 (Ro) (B) on the 665/615 ratio in 1536-well format are shown. Low 665/615 nm values represent higher intracellular cAMP levels. Results indicated that C6 cells (R201C mutation) had higher cAMP levels, and that 1,000–3,000 cells and 100 µM Ro-20-1724 were ideal for the assay to be performed in 1536-well format.
(TIF)

Figure S6 **Inhibition and Activation of Adenylyl Cyclase Activity.** Adenylyl Cyclase Inhibition and Activation. The effect of adenylyl cyclase inhibitors (A–D) and activator (E) were tested in C6 cells (expressing the R201C G_s_α). The effect of different adenylyl cyclase inhibitors ddA (2′,5′-dideoxyadenosine), KH (KH7), (E)-2-(1H-Benzo[d]imidazol-2-ylthio)-N′-(5-bromo-2-hydroxybenzylidene) propanehydrazide), MDL (MDL-12,330A), and SQ (SQ 22,536), at concentrations and time indicated were tested for effects on cAMP levels. Cells were also treated with the adenylyl cyclase activator Fsk (forskolin) (E) for 30 minutes. cAMP levels in C6 cells can be inhibited and stimulated in a time- and dose-dependent manner and were thus useful in screening for inhibitory and stimulatory molecules.
(TIF)

Figure S7 **Probenecid-Responsive cAMP Transport in CHO Cells.** The effect of probencid on extracellular (A) and intracellular (B) cAMP in WT and C6 mutant-transfected CHO cells was assessed. The concentration of probenecid is indicated. A decrease in the 666/615 ratio indicates an increase in cAMP. Depicted is the fact probenecid can decrease CHO cell cAMP transport.
(TIF)

Figure S8 **DMSO test plate.** The plate map for 1536-well screening format (A). Column 1 = CHO-WT treated with 0.77% DMSO control, column 2 = CHO-C6 with 0.77% DMSO control, column 3 = CHO-C6 with 76.7 µM ddA, column 4 = CHO-C6 with 76.7 µM forskolin and columns 5–48 = CHO-C6 treated with 0.77% DMSO. (B). Scatter plot of the results from a DMSO plate test in 1536-well format.
(TIF)

Figure S9 **A. Screen Top Confirmed Hit A. LOPAC Screen Top Confirmed Hit A** The effects of selected compounds tested in the LOPAC screen with various curve class responses as listed are shown. The structure of niclosamide, an anthelmintic, one of the most active compounds, is shown. **B. Screen Top Confirmed Hit B. LOPAC Screen Top Confirmed Hit B** The effects of selected compounds tested in the LOPAC screen with various curve class responses as listed are shown. The structure of tryphostin A9, Inhibitor of calcium release-activated calcium channels, and a selective inhibitor of PDGF receptor tyrosine kinase, is shown. **C. LOPAC Screen Top Confirmed Hit C.** The effects of selected compounds tested in the LOPAC screen with various curve class responses as listed are shown. The structure WIN 62,577, a non-peptide NK1 tachykinin receptor antagonist is shown.
(TIF)

Figure S10 **Forskolin dose response.** Six different cell lines stably transfected with Gsα [wild type (WT9), R201C mutants (C6, C7), and R201H mutants (H25, H37, H40)] were tested for a cAMP response to forskolin. cAMP was measured in a HTRF assay (see Methods). The lower the 665/590 ratio, the higher the cAMP concentration. The robust response of WT9 cells indicted that when treated with a suboptimal dose of forskolin it was a suitable line for testing the ability of compounds to inhibit G_s_α activity.
(TIF)

Table S1 **LOPAC Screen Assay Protocol.**
(TIF)

Table S2 **LOPAC Screen Curve Class Definitions.**
(TIF)

Table S3 **LOPAC Screen Curve Class Activity Summary.**
(TIF)

Table S4 **A. LOPAC Screen Cherry Pick Criteria. B. LOPAC Screen Cherry Pick Results.**
(TIF)

Table S5 **Cluster Analysis Compounds with molecule identifier, structure, IC50, and active link to a full description of the molecule in PubChem.**
(PDF)
