# Supplementary figures and images for: A High Throughput Screening Assay System for the Identification of Small Molecule Inhibitors of gsp
Source: PLoS One. 2014 Mar 25;9(3):e90766. doi: 10.1371/journal.pone.0090766 (PMC3965391; doi:10.1371/journal.pone.0090766)

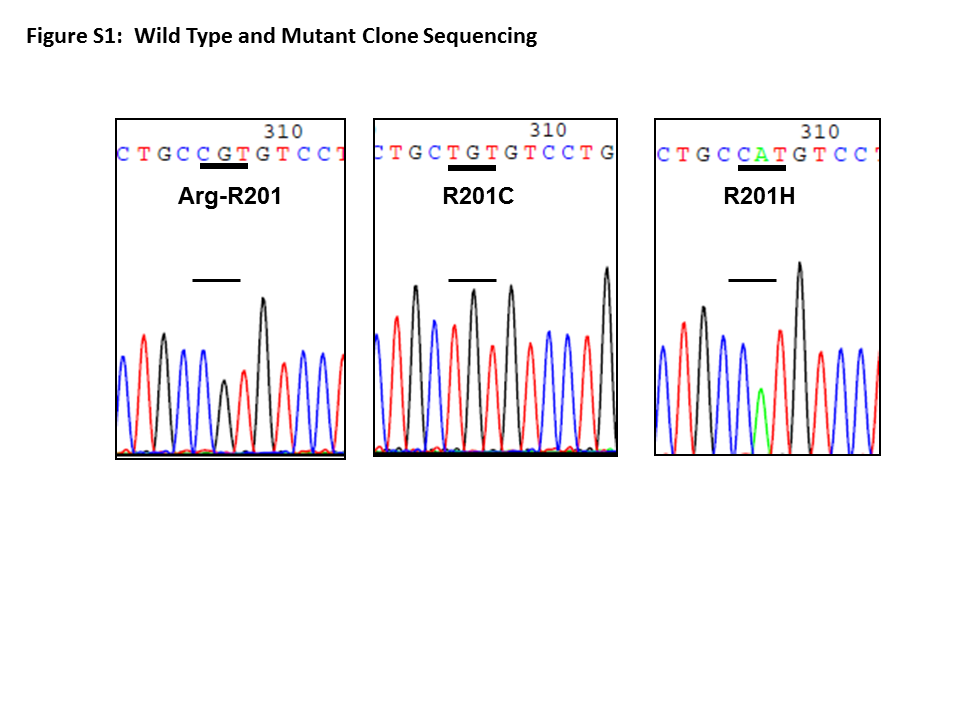

Supplement: Figure S1 — Wild Type and Mutant Clone Sequencing. Wild type and mutant Gsα clone sequencing. Recombinant plasmids carrying the WT (Arg201), Cys (R201C) and His (R201H) Gsα were sequenced using an internal oligonucleotide to confirm the mutated area. Sequences from the pertinent area are shown. (TIF) [file pone.0090766.s001.tif]

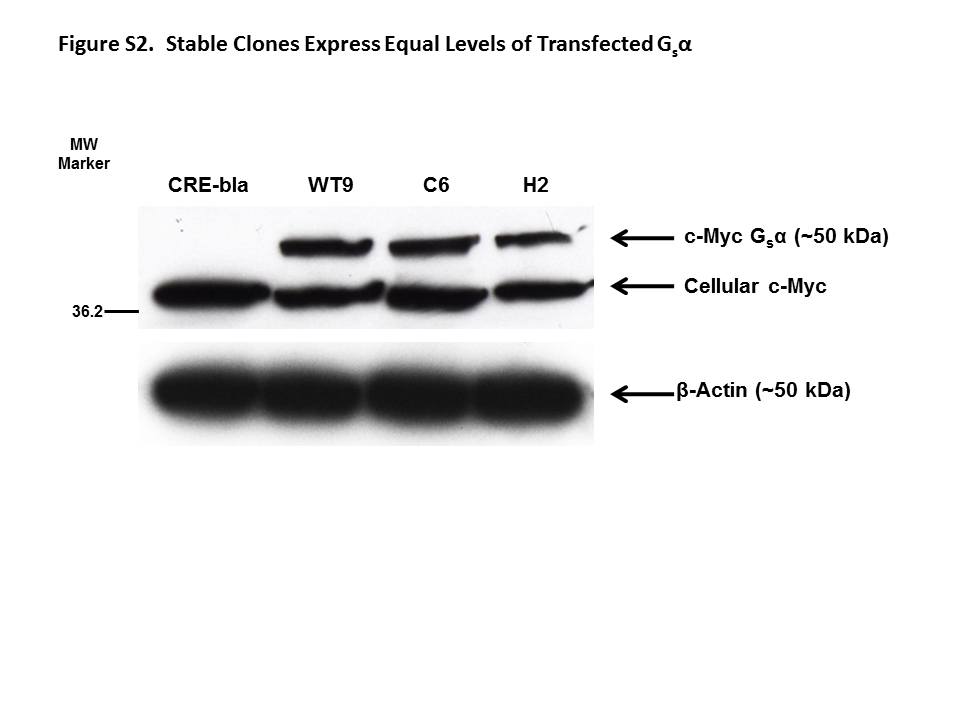

Supplement: Figure S2 — Stable Clones Express Equal Levels of Transfected Gsα. Equal Levels of Transfected Gsα Expression. Mutant and WT transfected Gsα was tagged with c-Myc to assess transfection efficiency. Cell lines were chosen that demonstrated equal amounts of c-Myc expression, reflecting equal transfected Gsα expression. Cellular c-Myc, a molecular weight indicator and β-actin (loading control) are also labeled. (TIF) [file pone.0090766.s002.tif]

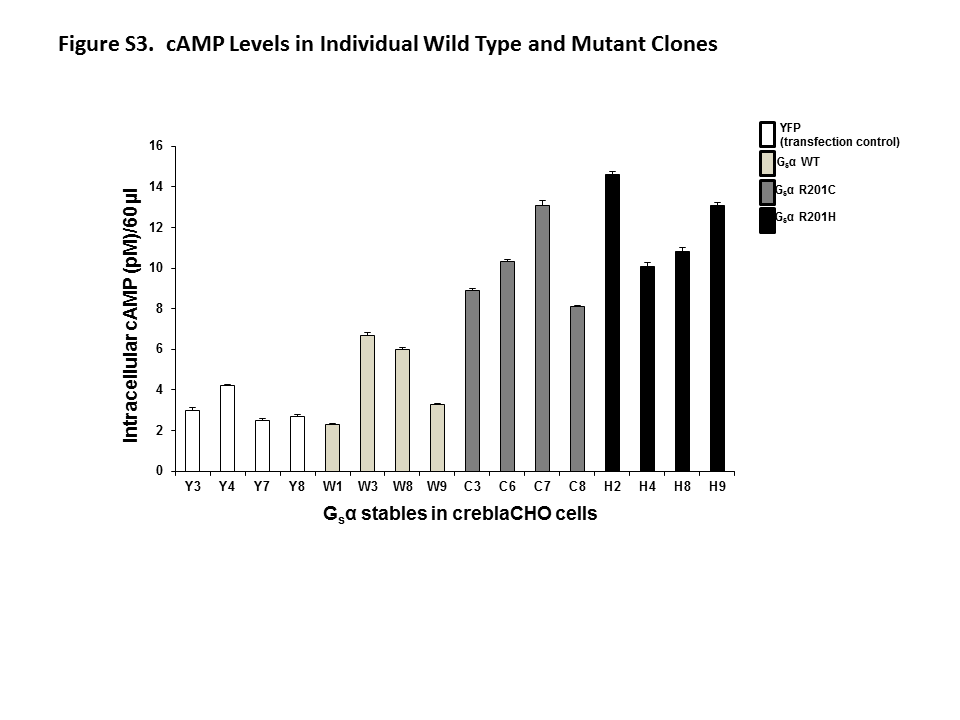

Supplement: Figure S3 — cAMP Levels in Individual Wild Type and Mutant Clones. Wild Type and Mutant Cell Line Performance. cAMP levels from individual stable clones expressing the YFP-N1 (control cells; Y, open bars), WT Gsα (W, light grey bars), R201C Gsα (C; dark grey bars) and R201H Gsα (H, black bars) were measured using a cAMP ELISA assay. Assays were performed in triplicate and repeated at least three times. (TIF) [file pone.0090766.s003.tif]

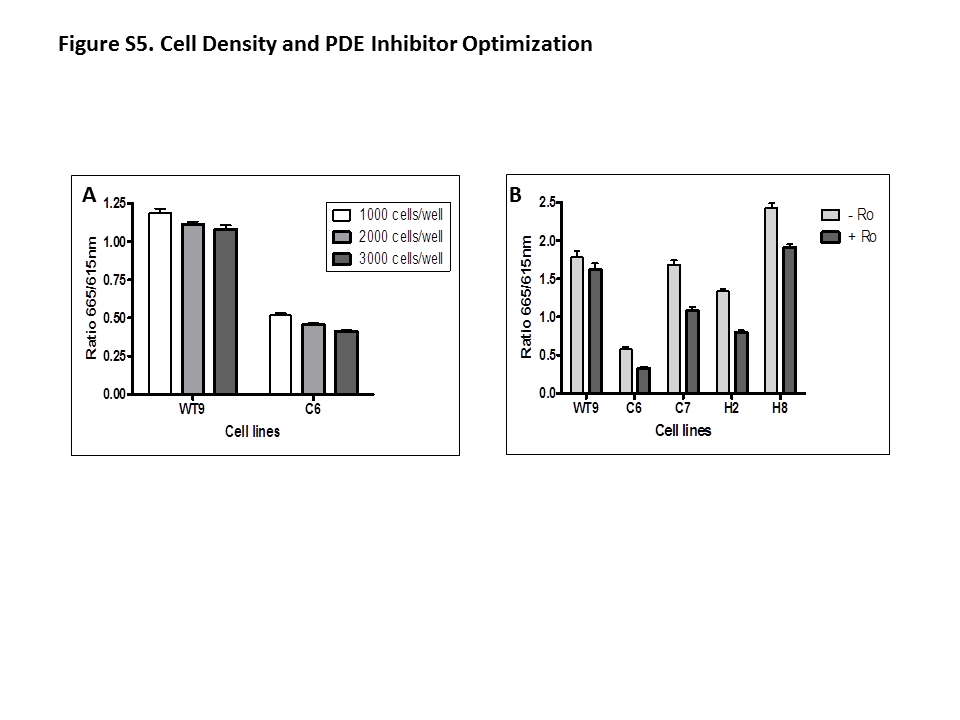

Supplement: Figure S5 — Cell Density and PDE Inhibitor Optimization. The effect of cell density (A) and the phosphodiesterase inhibitor Ro-20-1724 (Ro) (B) on the 665/615 ratio in 1536-well format are shown. Low 665/615 nm values represent higher intracellular cAMP levels. Results indicated that C6 cells (R201C mutation) had higher cAMP levels, and that 1,000–3,000 cells and 100 µM Ro-20-1724 were ideal for the assay to be performed in 1536-well format. (TIF) [file pone.0090766.s005.tif]

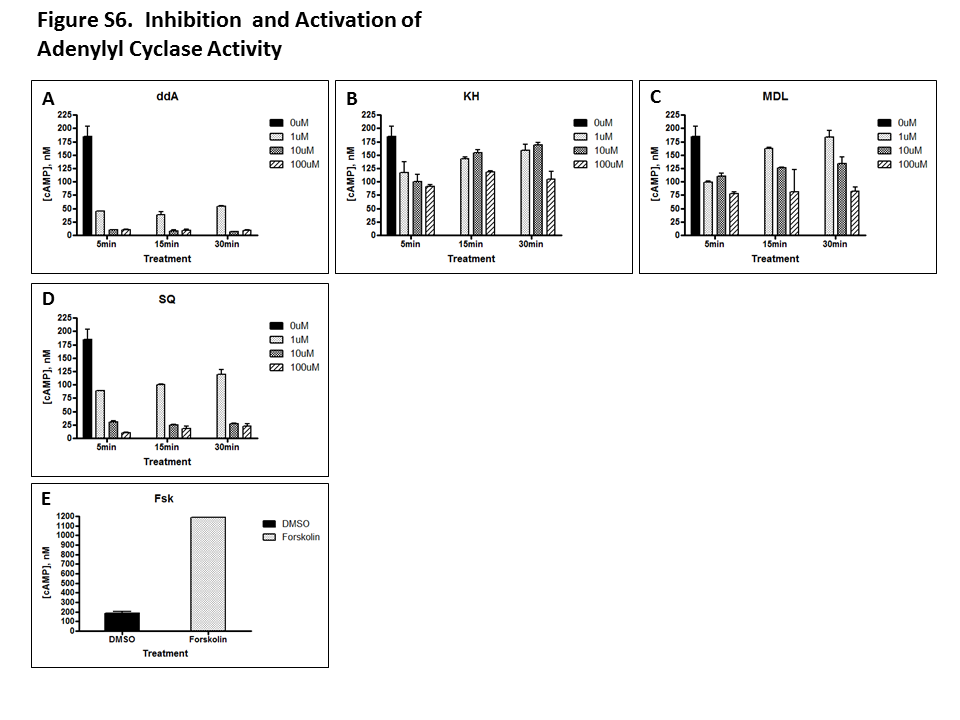

Supplement: Figure S6 — Inhibition and Activation of Adenylyl Cyclase Activity. Adenylyl Cyclase Inhibition and Activation. The effect of adenylyl cyclase inhibitors (A–D) and activator (E) were tested in C6 cells (expressing the R201C Gsα). The effect of different adenylyl cyclase inhibitors ddA (2′,5′-dideoxyadenosine), KH (KH7), (E)-2-(1H-Benzo[d]imidazol-2-ylthio)-N′-(5-bromo-2-hydroxybenzylidene) propanehydrazide), MDL (MDL-12,330A), and SQ (SQ 22,536), at concentrations and time indicated were tested for effects on cAMP levels. Cells were also treated with the adenylyl cyclase activator Fsk (forskolin) (E) for 30 minutes. cAMP levels in C6 cells can be inhibited and stimulated in a time- and dose-dependent manner and were thus useful in screening for inhibitory and stimulatory molecules. (TIF) [file pone.0090766.s006.tif]

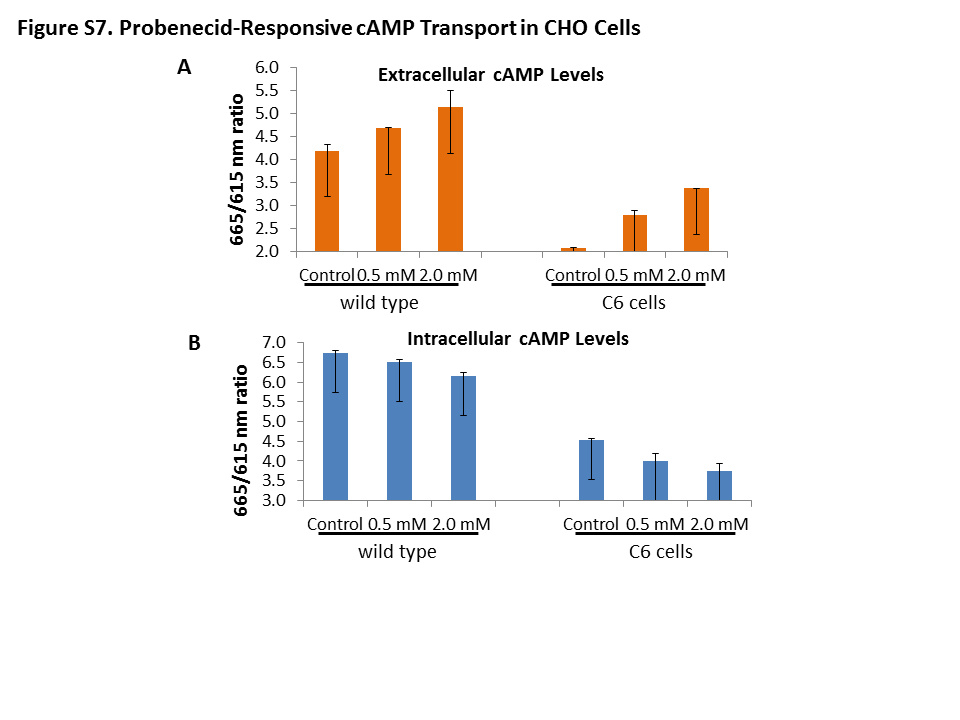

Supplement: Figure S7 — Probenecid-Responsive cAMP Transport in CHO Cells. The effect of probencid on extracellular (A) and intracellular (B) cAMP in WT and C6 mutant-transfected CHO cells was assessed. The concentration of probenecid is indicated. A decrease in the 666/615 ratio indicates an increase in cAMP. Depicted is the fact probenecid can decrease CHO cell cAMP transport. (TIF) [file pone.0090766.s007.tif]

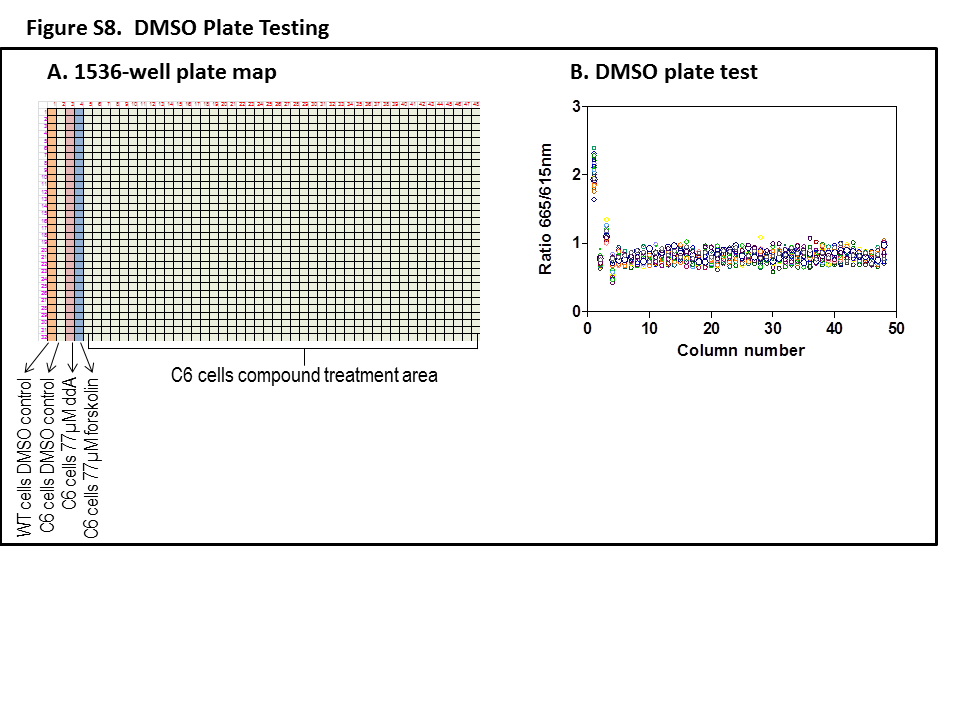

Supplement: Figure S8 — DMSO test plate. The plate map for 1536-well screening format (A). Column 1 = CHO-WT treated with 0.77% DMSO control, column 2 = CHO-C6 with 0.77% DMSO control, column 3 = CHO-C6 with 76.7 µM ddA, column 4 = CHO-C6 with 76.7 µM forskolin and columns 5–48 = CHO-C6 treated with 0.77% DMSO. (B). Scatter plot of the results from a DMSO plate test in 1536-well format. (TIF) [file pone.0090766.s008.tif]

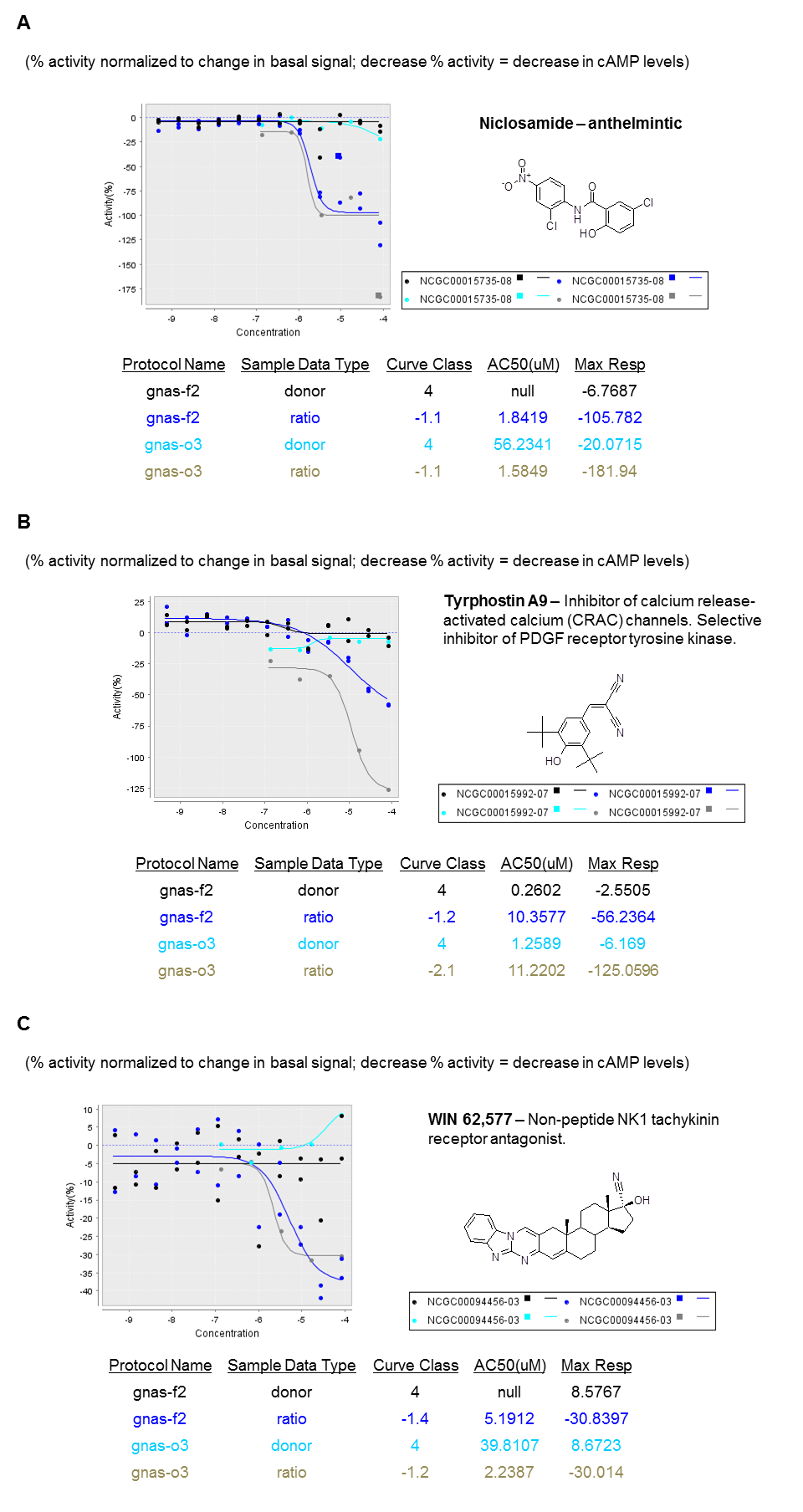

Supplement: Figure S9 — A. Screen Top Confirmed Hit A. LOPAC Screen Top Confirmed Hit A The effects of selected compounds tested in the LOPAC screen with various curve class responses as listed are shown. The structure of niclosamide, an anthelmintic, one of the most active compounds, is shown. B. Screen Top Confirmed Hit B. LOPAC Screen Top Confirmed Hit B The effects of selected compounds tested in the LOPAC screen with various curve class responses as listed are shown. The structure of tryphostin A9, Inhibitor of calcium release-activated calcium channels, and a selective inhibitor of PDGF receptor tyrosine kinase, is shown. C. LOPAC Screen Top Confirmed Hit C. The effects of selected compounds tested in the LOPAC screen with various curve class responses as listed are shown. The structure WIN 62,577, a non-peptide NK1 tachykinin receptor antagonist is shown. (TIF) [file pone.0090766.s009.tif]

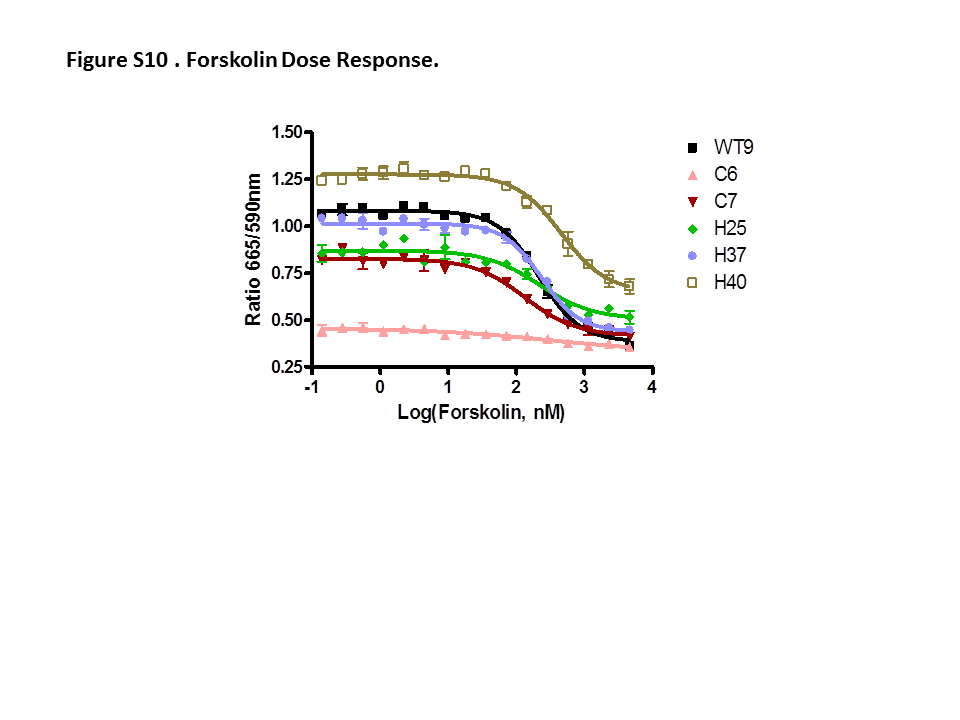

Supplement: Figure S10 — Forskolin dose response. Six different cell lines stably transfected with Gsα [wild type (WT9), R201C mutants (C6, C7), and R201H mutants (H25, H37, H40)] were tested for a cAMP response to forskolin. cAMP was measured in a HTRF assay (see Methods). The lower the 665/590 ratio, the higher the cAMP concentration. The robust response of WT9 cells indicted that when treated with a suboptimal dose of forskolin it was a suitable line for testing the ability of compounds to inhibit Gsα activity. (TIF) [file pone.0090766.s010.tif]

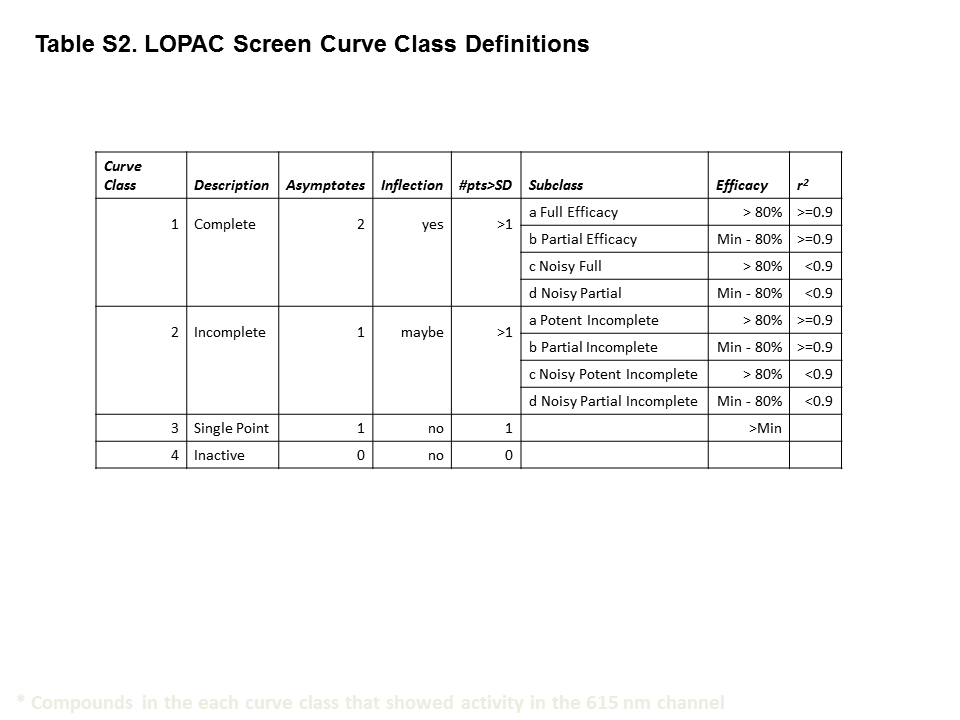

Supplement: Table S2 — LOPAC Screen Curve Class Definitions. (TIF) [file pone.0090766.s012.tif]
