# Supplementary material for: A High Throughput Screening Assay System for the Identification of Small Molecule Inhibitors of gsp
Source: PLoS One. 2014 Mar 25;9(3):e90766. doi: 10.1371/journal.pone.0090766 (PMC3965391; doi:10.1371/journal.pone.0090766)
Supplement: Table S5 — Cluster Analysis Compounds with molecule identifier, structure, IC50, and active link to a full description of the molecule in PubChem. (PDF) [file pone.0090766.s015.pdf]

Sample ID	Cluster	Members	Structure	IC50(uM)	PubChem link
MLS001215208-01	1	27	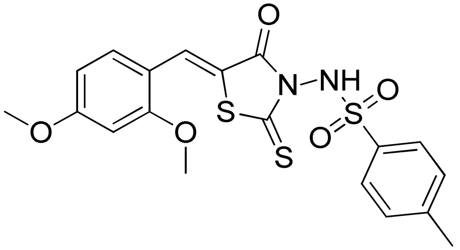	7.9433	http://pubchem.ncbi.nlm.nih.gov/summary/summary.cgi?sid=49666882
MLS001210478-01	1	27	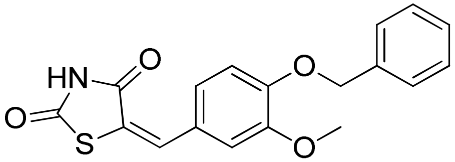	3.1623	http://pubchem.ncbi.nlm.nih.gov/summary/summary.cgi?sid=49643895
MLS000778429-01	1	27	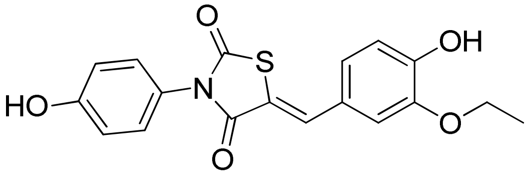	5.6234	http://pubchem.ncbi.nlm.nih.gov/summary/summary.cgi?sid=26663362
MLS000755805-01	1	27	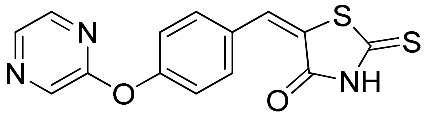	7.9433	http://pubchem.ncbi.nlm.nih.gov/summary/summary.cgi?sid=24824463
MLS000850781-01	1	27	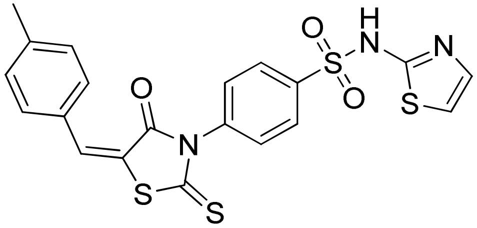	10	http://pubchem.ncbi.nlm.nih.gov/summary/summary.cgi?sid=26731521

MLS001215276-01	1	27	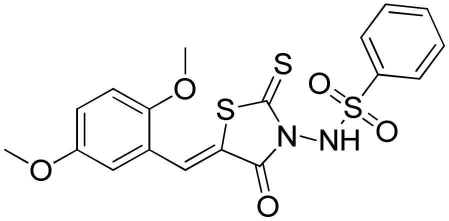	5.0119	http://pubchem.ncbi.nlm.nih.gov/summary/summary.cgi?sid=49646959
MLS000686304-01	1	27	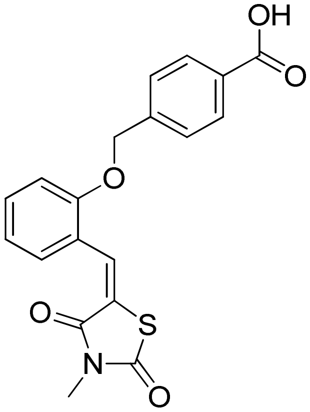	8.9125	http://pubchem.ncbi.nlm.nih.gov/summary/summary.cgi?sid=24822909
MLS000779147-01	1	27	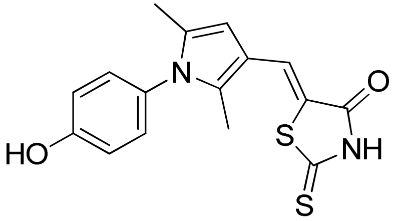	25.1189	http://pubchem.ncbi.nlm.nih.gov/summary/summary.cgi?sid=26660292
MLS001215303-01	1	27	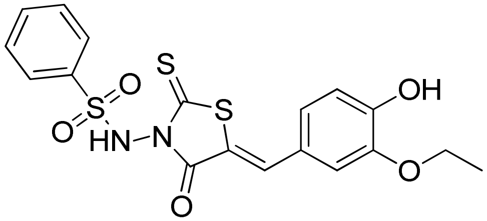	39.8107	http://pubchem.ncbi.nlm.nih.gov/summary/summary.cgi?sid=49646131

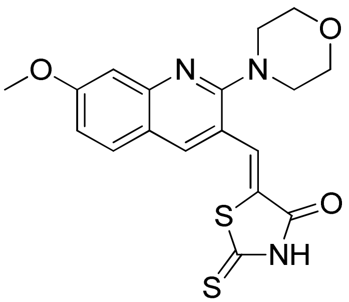

MLS000590439-01	1	27	2.5119	http://pubchem.ncbi.nlm.nih.gov/summary/summary.cgi?sid=17513514

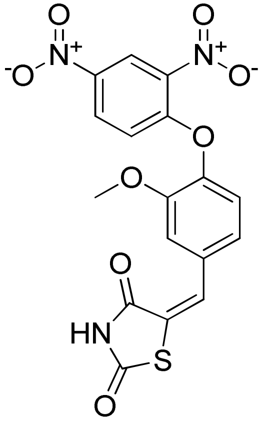

MLS000765074-01	1	27	5.6234	http://pubchem.ncbi.nlm.nih.gov/summary/summary.cgi?sid=24822652

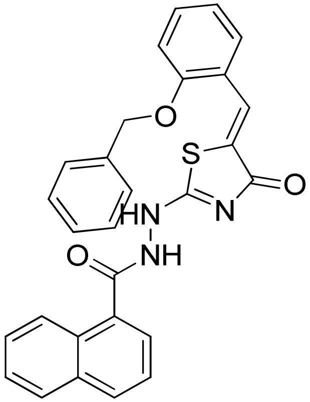

MLS000566525-01	1	27	11.2202	http://pubchem.ncbi.nlm.nih.gov/summary/summary.cgi?sid=17408099

MLS000850782-01	1	27	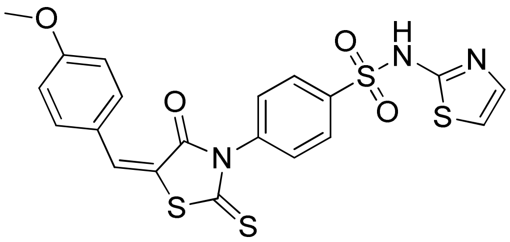	11.2202	http://pubchem.ncbi.nlm.nih.gov/summary/summary.cgi?sid=26731479
MLS000335351-01	1	27	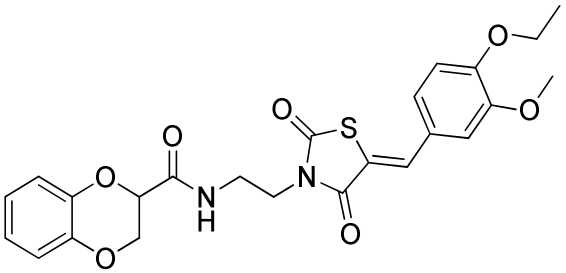	14.1254	http://pubchem.ncbi.nlm.nih.gov/summary/summary.cgi?sid=22409231
MLS000333439-01	1	27	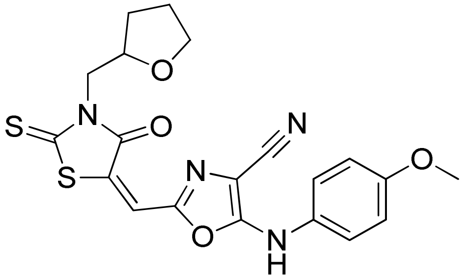	22.3872	http://pubchem.ncbi.nlm.nih.gov/summary/summary.cgi?sid=26671258
MLS000711080-01	1	27	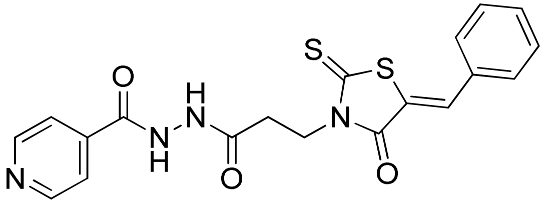	12.5893	http://pubchem.ncbi.nlm.nih.gov/summary/summary.cgi?sid=24784043

MLS001214710-01	1	27	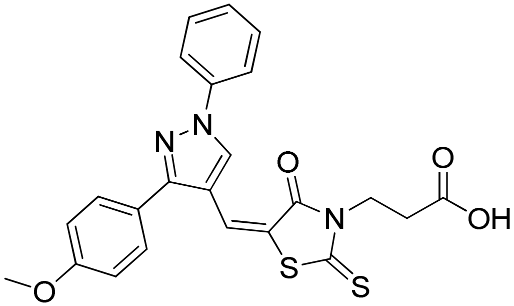	3.1623	http://pubchem.ncbi.nlm.nih.gov/summary/summary.cgi?sid=49646097
MLS000703572-01	1	27	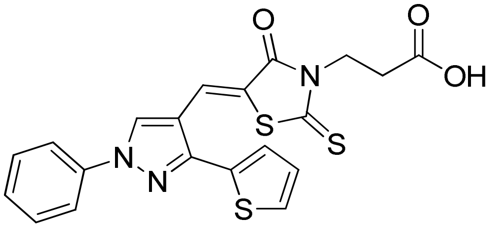	4.4668	http://pubchem.ncbi.nlm.nih.gov/summary/summary.cgi?sid=24801573
MLS000331466-01	1	27	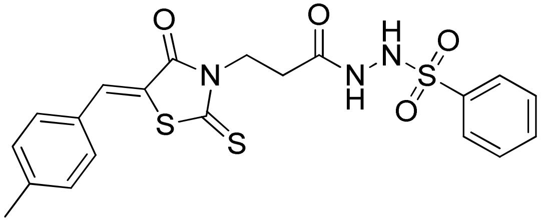	31.6228	http://pubchem.ncbi.nlm.nih.gov/summary/summary.cgi?sid=24791215
MLS000775324-01	1	27	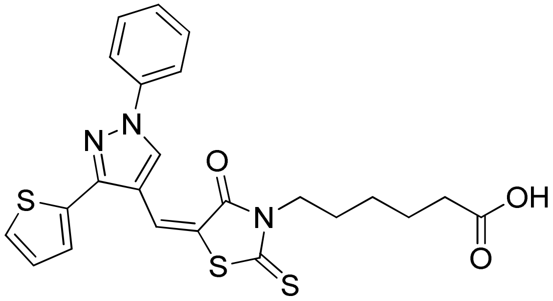	35.4813	http://pubchem.ncbi.nlm.nih.gov/summary/summary.cgi?sid=24836019

MLS002633376-01	1	27	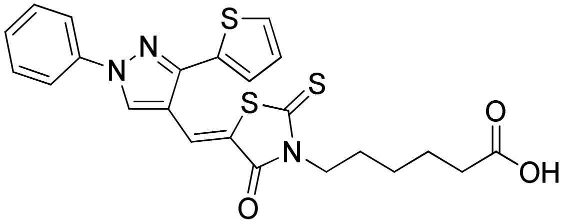	39.8107	http://pubchem.ncbi.nlm.nih.gov/summary/summary.cgi?sid=89852067
MLS001034529-01	1	27	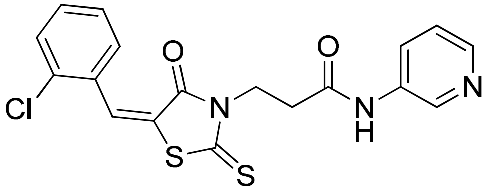	6.3096	http://pubchem.ncbi.nlm.nih.gov/summary/summary.cgi?sid=49816355
MLS001157953-01	1	27	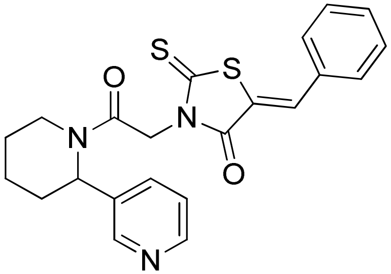	17.7828	http://pubchem.ncbi.nlm.nih.gov/summary/summary.cgi?sid=49822477
MLS001157871-01	1	27	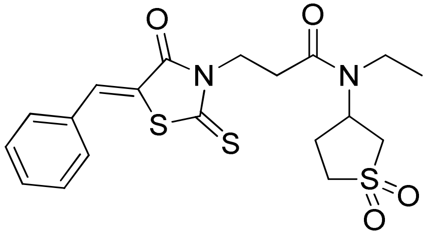	28.1838	http://pubchem.ncbi.nlm.nih.gov/summary/summary.cgi?sid=49822511

MLS001171467-01	1	27	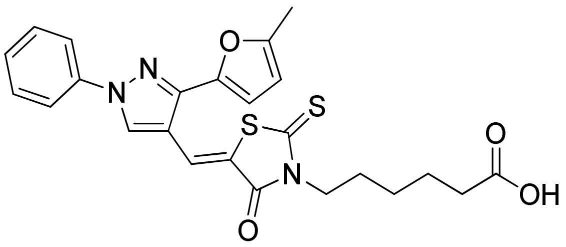	31.6228	http://pubchem.ncbi.nlm.nih.gov/summary/summary.cgi?sid=49673957
MLS001163553-01	1	27	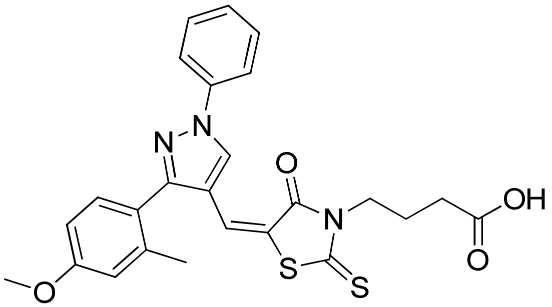	11.2202	http://pubchem.ncbi.nlm.nih.gov/summary/summary.cgi?sid=47196686
MLS001215512-01	1	27	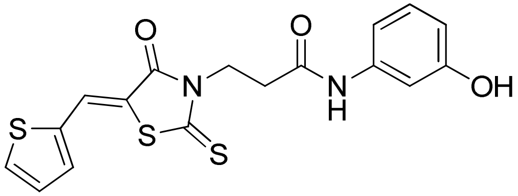	22.3872	http://pubchem.ncbi.nlm.nih.gov/summary/summary.cgi?sid=49726708
MLS000912277-01	2	14	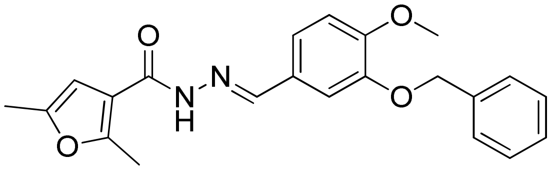	10	http://pubchem.ncbi.nlm.nih.gov/summary/summary.cgi?sid=49714058
MLS001146490-01	2	14	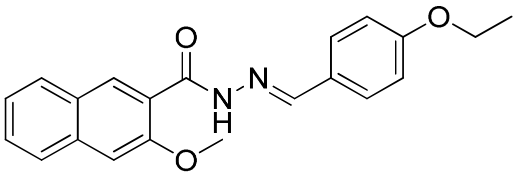	28.1838	http://pubchem.ncbi.nlm.nih.gov/summary/summary.cgi?sid=49821204

MLS001139341-01	2	14	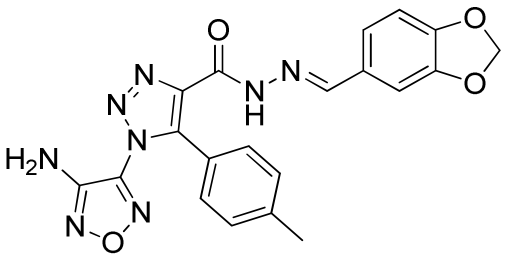	8.9125	http://pubchem.ncbi.nlm.nih.gov/summary/summary.cgi?sid=49819872
MLS001139448-01	2	14	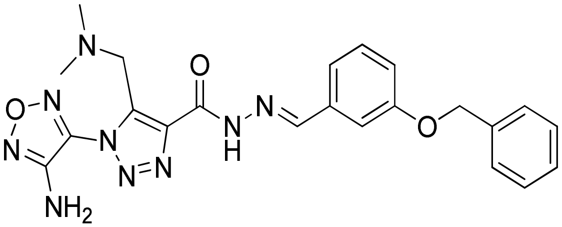	3.5481	http://pubchem.ncbi.nlm.nih.gov/summary/summary.cgi?sid=49820327
MLS001142840-01	2	14	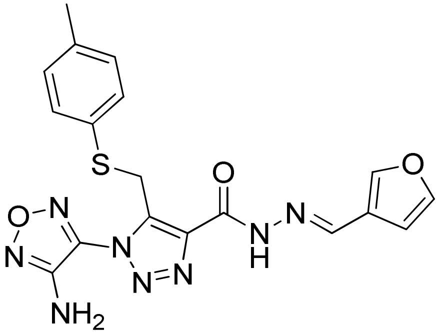	8.9125	http://pubchem.ncbi.nlm.nih.gov/summary/summary.cgi?sid=49820227
MLS001142666-01	2	14	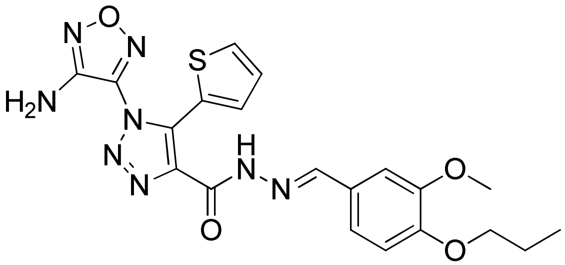	10	http://pubchem.ncbi.nlm.nih.gov/summary/summary.cgi?sid=49829167

MLS000950063-01	2	14	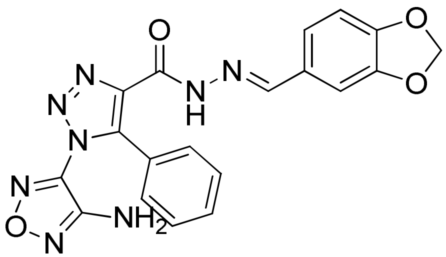	15.8489	http://pubchem.ncbi.nlm.nih.gov/summary/summary.cgi?sid=49715853
MLS000391853-01	2	14	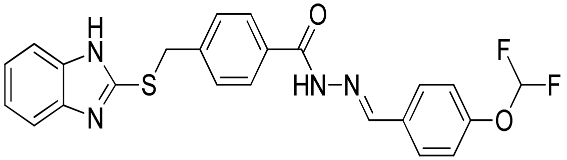	15.8489	http://pubchem.ncbi.nlm.nih.gov/summary/summary.cgi?sid=22409883
MLS001223916-01	2	14	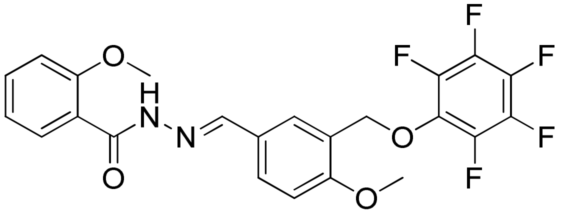	17.7828	http://pubchem.ncbi.nlm.nih.gov/summary/summary.cgi?sid=49823934
MLS001125728-01	2	14	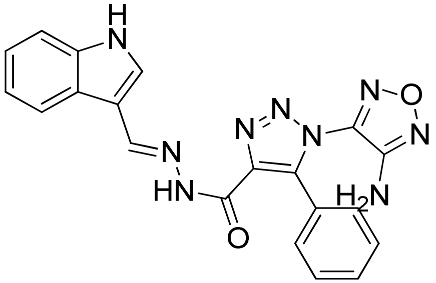	17.7828	http://pubchem.ncbi.nlm.nih.gov/summary/summary.cgi?sid=49825727

MLS001142665-01	2	14	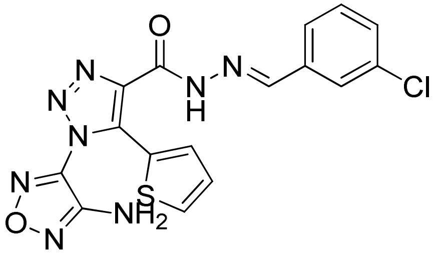	22.3872	http://pubchem.ncbi.nlm.nih.gov/summary/summary.cgi?sid=49828660
MLS001142684-01	2	14	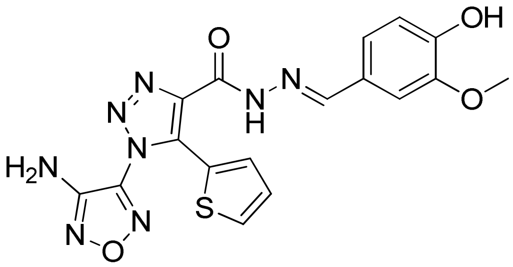	31.6228	http://pubchem.ncbi.nlm.nih.gov/summary/summary.cgi?sid=49829392
MLS001142866-01	2	14	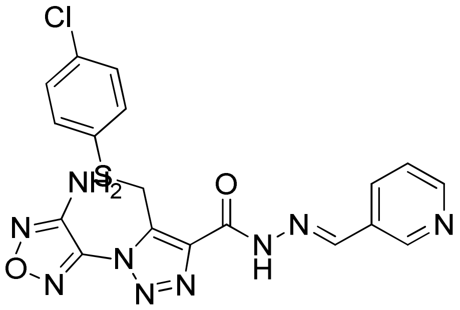	35.4813	http://pubchem.ncbi.nlm.nih.gov/summary/summary.cgi?sid=49820234
MLS001142659-01	2	14	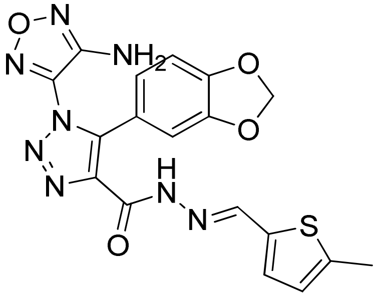	35.4813	http://pubchem.ncbi.nlm.nih.gov/summary/summary.cgi?sid=49819819

MLS000032897-01	3	14	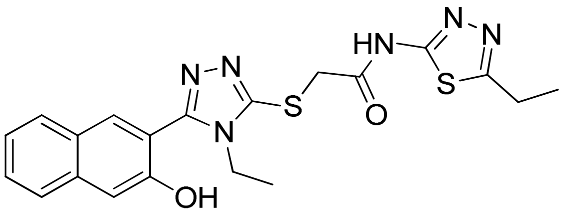	7.9433	http://pubchem.ncbi.nlm.nih.gov/summary/summary.cgi?sid=843972
MLS000121713-01	3	14	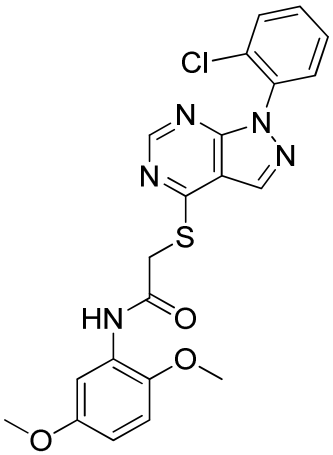	0.8913	http://pubchem.ncbi.nlm.nih.gov/summary/summary.cgi?sid=14721552
MLS000581831-01	3	14	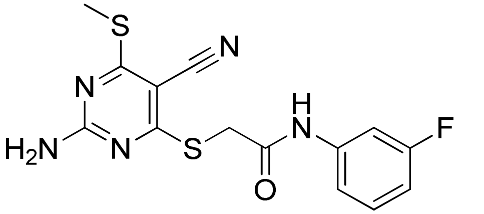	3.9811	http://pubchem.ncbi.nlm.nih.gov/summary/summary.cgi?sid=17504754
MLS000026902-01	3	14	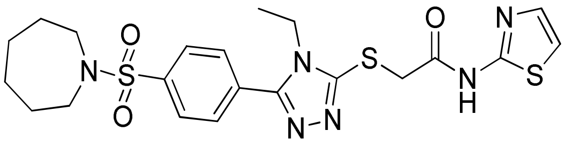	8.9125	http://pubchem.ncbi.nlm.nih.gov/summary/summary.cgi?sid=16953352

MLS000120988-01	3	14	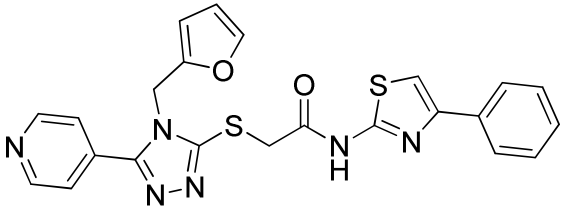	3.5481	http://pubchem.ncbi.nlm.nih.gov/summary/summary.cgi?sid=16953298
MLS000581485-01	3	14	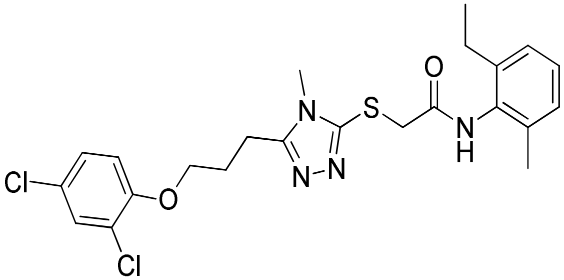	7.0795	http://pubchem.ncbi.nlm.nih.gov/summary/summary.cgi?sid=17433424
MLS000538142-01	3	14	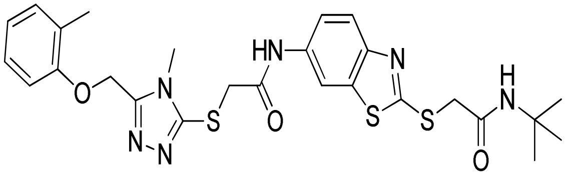	14.1254	http://pubchem.ncbi.nlm.nih.gov/summary/summary.cgi?sid=17386392
MLS000391488-01	3	14	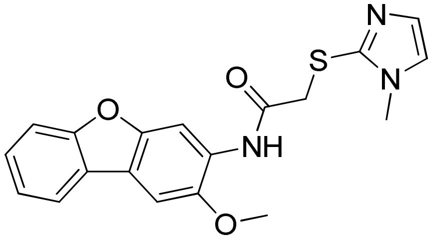	15.8489	http://pubchem.ncbi.nlm.nih.gov/summary/summary.cgi?sid=22408416
MLS000073611-01	3	14	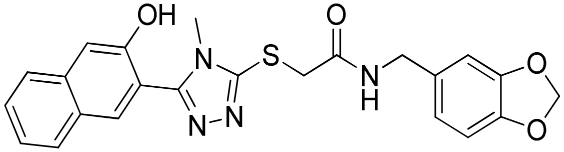	19.9526	http://pubchem.ncbi.nlm.nih.gov/summary/summary.cgi?sid=850865

MLS001223062-01	3	14	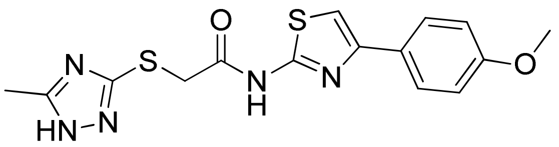	22.3872	http://pubchem.ncbi.nlm.nih.gov/summary/summary.cgi?sid=49728088
MLS000878596-01	3	14	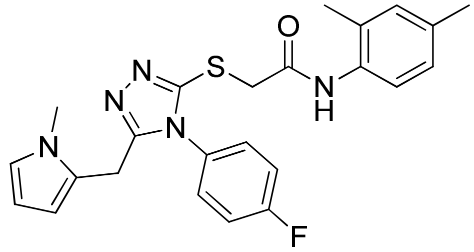	28.1838	http://pubchem.ncbi.nlm.nih.gov/summary/summary.cgi?sid=24838415
MLS000098934-01	3	14	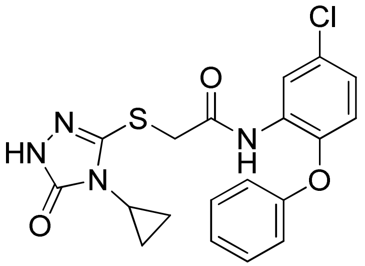	31.6228	http://pubchem.ncbi.nlm.nih.gov/summary/summary.cgi?sid=3716263
MLS000077078-01	3	14	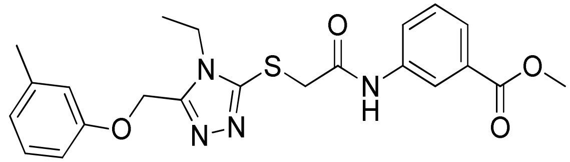	39.8107	http://pubchem.ncbi.nlm.nih.gov/summary/summary.cgi?sid=852486
MLS001222970-01	3	14	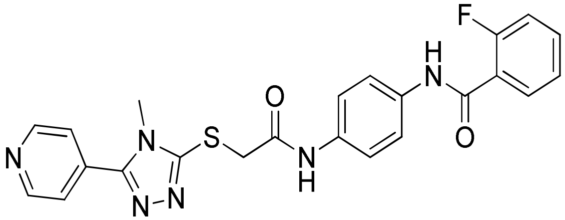	50.1187	http://pubchem.ncbi.nlm.nih.gov/summary/summary.cgi?sid=49727758

MLS000049694-01	4	13	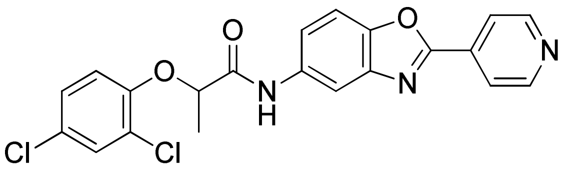	15.8489	http://pubchem.ncbi.nlm.nih.gov/summary/summary.cgi?sid=4260853
MLS002248306-01	4	13	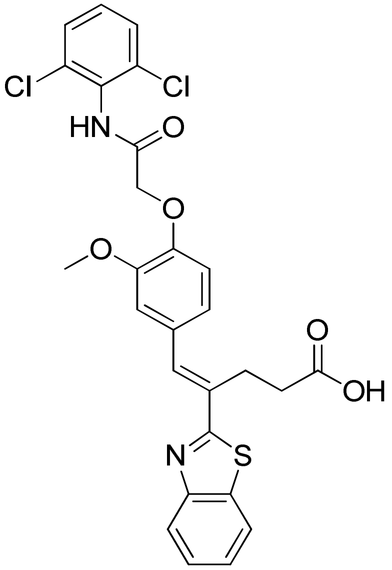	2.5119	http://pubchem.ncbi.nlm.nih.gov/summary/summary.cgi?sid=85268849
MLS002163313-01	4	13	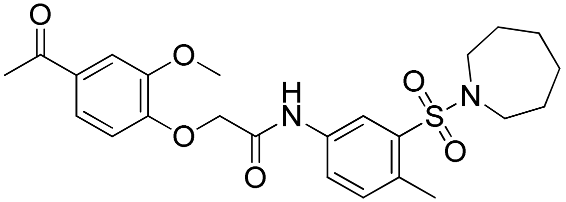	3.5481	http://pubchem.ncbi.nlm.nih.gov/summary/summary.cgi?sid=57259665
MLS002158990-01	4	13	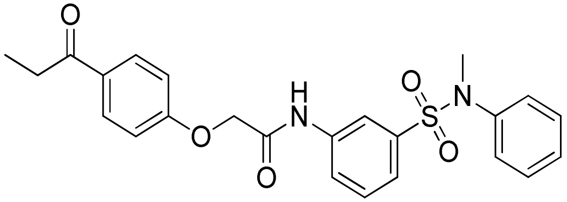	3.9811	http://pubchem.ncbi.nlm.nih.gov/summary/summary.cgi?sid=57263130

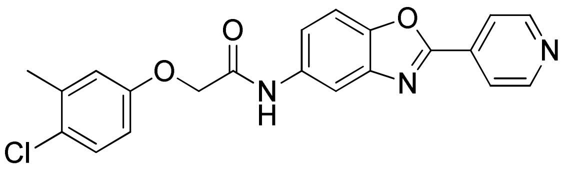

MLS000040007-01	4	13		35.4813	http://pubchem.ncbi.nlm.nih.gov/summary/summary.cgi?sid=861282

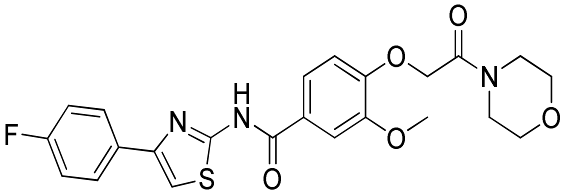

MLS000334344-01	4	13		12.5893	http://pubchem.ncbi.nlm.nih.gov/summary/summary.cgi?sid=22408888

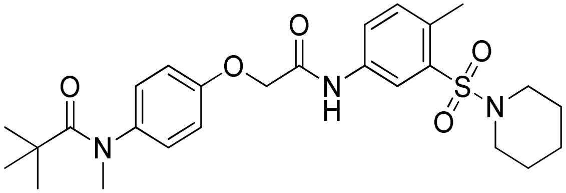

MLS002252318-01	4	13		17.7828	http://pubchem.ncbi.nlm.nih.gov/summary/summary.cgi?sid=85198345

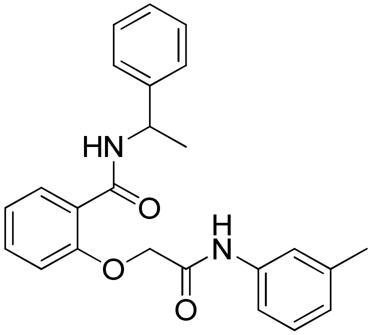

MLS001162588-01	4	13		22.3872	http://pubchem.ncbi.nlm.nih.gov/summary/summary.cgi?sid=49826088

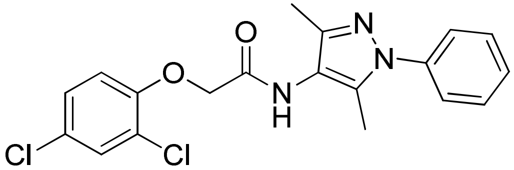

MLS000394578-01	4	13		22.3872	http://pubchem.ncbi.nlm.nih.gov/summary/summary.cgi?sid=22407316

MLS002157773-01	4	13	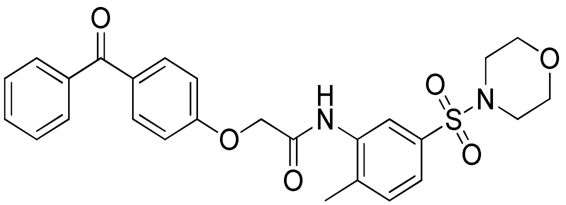	28.1838	http://pubchem.ncbi.nlm.nih.gov/summary/summary.cgi?sid=57266521
MLS000530891-01	4	13	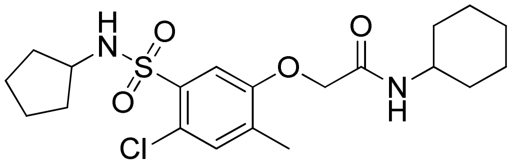	35.4813	http://pubchem.ncbi.nlm.nih.gov/summary/summary.cgi?sid=14738855
MLS000084641-01	4	13	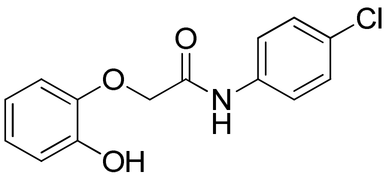	39.8107	http://pubchem.ncbi.nlm.nih.gov/summary/summary.cgi?sid=4247491
MLS002157282-01	4	13	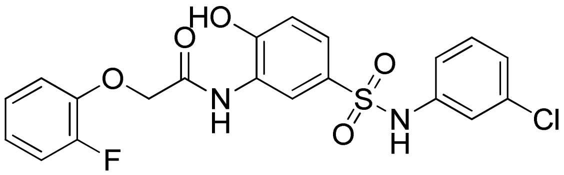	5.6234	http://pubchem.ncbi.nlm.nih.gov/summary/summary.cgi?sid=57263037
MLS001125049-01	5	12	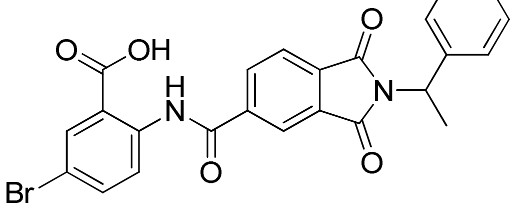	10	http://pubchem.ncbi.nlm.nih.gov/summary/summary.cgi?sid=49819363

MLS001181350-01	5	12	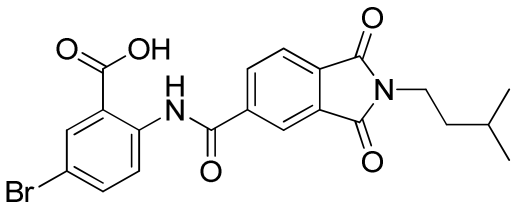	10	http://pubchem.ncbi.nlm.nih.gov/summary/summary.cgi?sid=47200585
MLS000394125-01	5	12	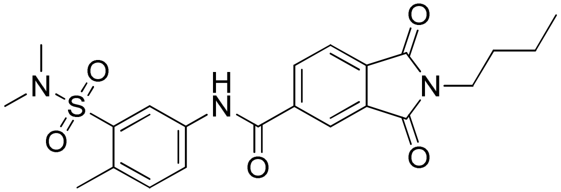	4.4668	http://pubchem.ncbi.nlm.nih.gov/summary/summary.cgi?sid=22410665
MLS001125011-01	5	12	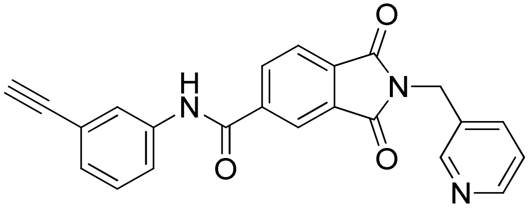	4.4668	http://pubchem.ncbi.nlm.nih.gov/summary/summary.cgi?sid=49723899
MLS000064902-01	5	12	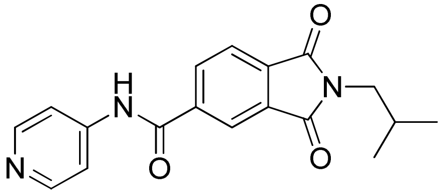	6.3096	http://pubchem.ncbi.nlm.nih.gov/summary/summary.cgi?sid=4258848

MLS000862940-01	5	12	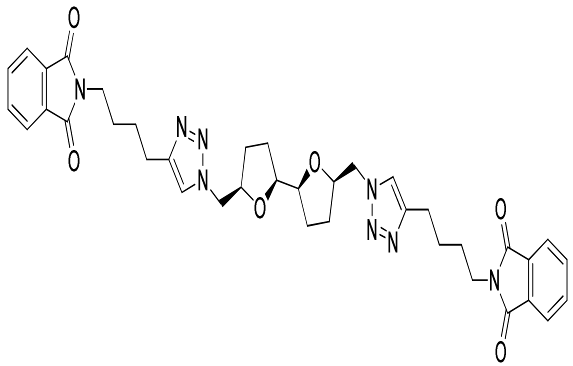	4.4668	http://pubchem.ncbi.nlm.nih.gov/summary/summary.cgi?sid=74373619
MLS001123926-01	5	12	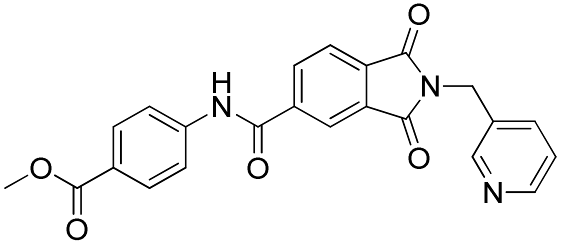	8.9125	http://pubchem.ncbi.nlm.nih.gov/summary/summary.cgi?sid=49723583
MLS001034698-01	5	12	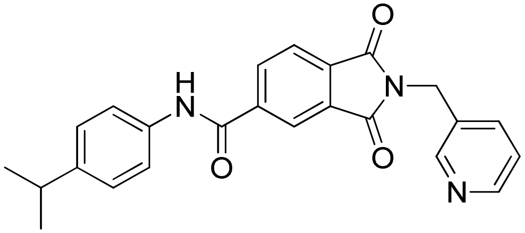	14.1254	http://pubchem.ncbi.nlm.nih.gov/summary/summary.cgi?sid=49732591
MLS001125015-01	5	12	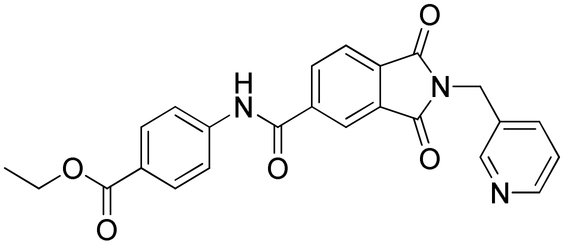	15.8489	http://pubchem.ncbi.nlm.nih.gov/summary/summary.cgi?sid=49723994

MLS001035359-01	5	12	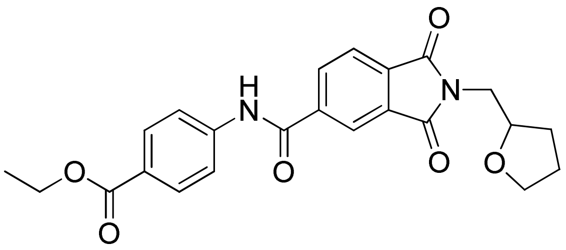	15.8489	http://pubchem.ncbi.nlm.nih.gov/summary/summary.cgi?sid=49816638
MLS000049852-01	5	12	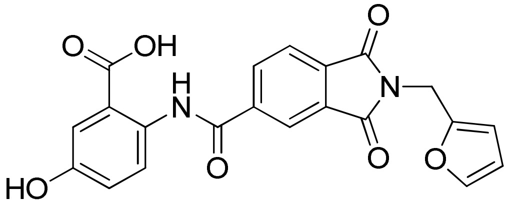	17.7828	http://pubchem.ncbi.nlm.nih.gov/summary/summary.cgi?sid=4263603
MLS000535216-01	5	12	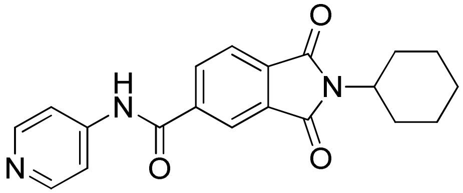	2.5119	http://pubchem.ncbi.nlm.nih.gov/summary/summary.cgi?sid=14731475
MLS000764737-01	6	11	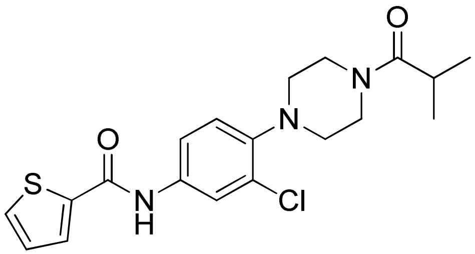	35.4813	http://pubchem.ncbi.nlm.nih.gov/summary/summary.cgi?sid=24807232

			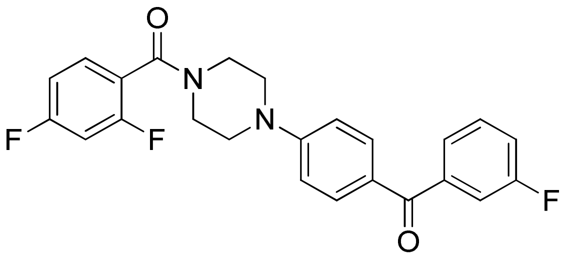		
MLS001124808-01	6	11		35.4813	http://pubchem.ncbi.nlm.nih.gov/summary/summary.cgi?sid=49723714
			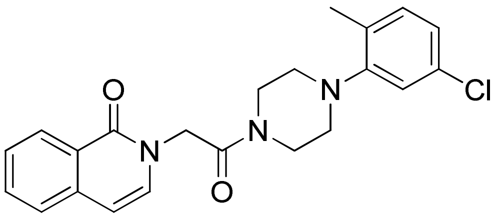		
MLS000086165-01	6	11		12.5893	http://pubchem.ncbi.nlm.nih.gov/summary/summary.cgi?sid=4240935
			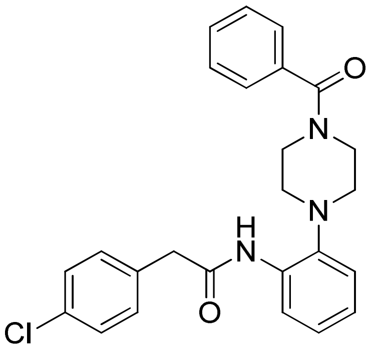		
MLS001034278-01	6	11		14.1254	http://pubchem.ncbi.nlm.nih.gov/summary/summary.cgi?sid=49816131
			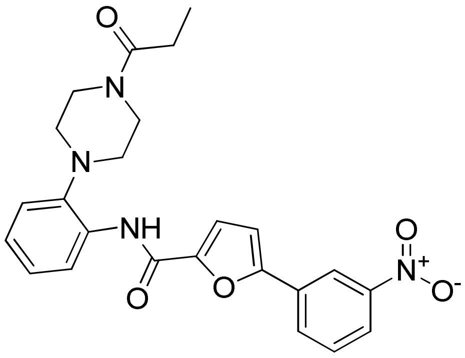		
MLS000579470-01	6	11		17.7828	http://pubchem.ncbi.nlm.nih.gov/summary/summary.cgi?sid=17506869

MLS000546853-01	6	11	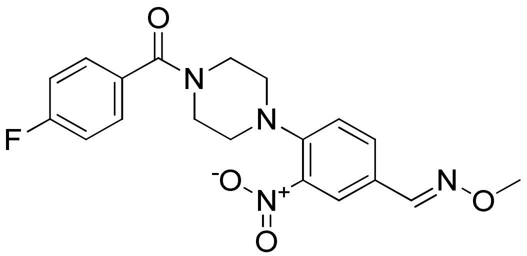	22.3872	http://pubchem.ncbi.nlm.nih.gov/summary/summary.cgi?sid=17409393
MLS001030525-01	6	11	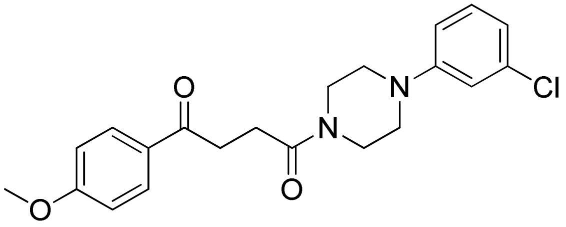	28.1838	http://pubchem.ncbi.nlm.nih.gov/summary/summary.cgi?sid=49734544
MLS001125755-01	6	11	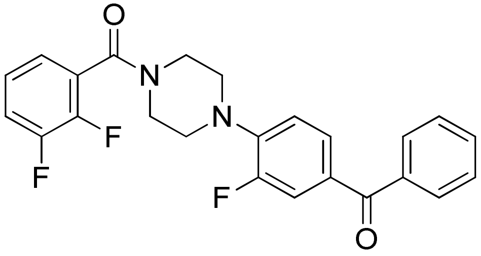	31.6228	http://pubchem.ncbi.nlm.nih.gov/summary/summary.cgi?sid=49724287
MLS002171379-01	6	11	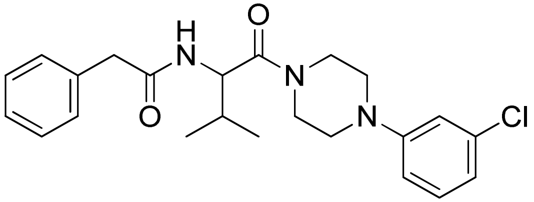	35.4813	http://pubchem.ncbi.nlm.nih.gov/summary/summary.cgi?sid=57263654
MLS000334486-01	6	11	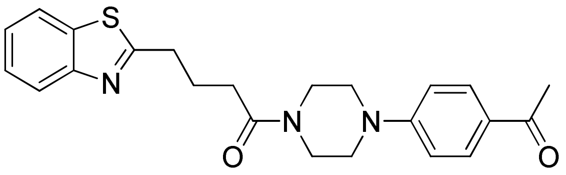	35.4813	http://pubchem.ncbi.nlm.nih.gov/summary/summary.cgi?sid=22410332

MLS000771283-01	6	11	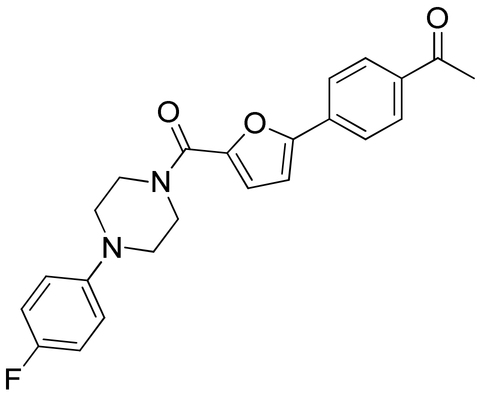	5.6234	http://pubchem.ncbi.nlm.nih.gov/summary/summary.cgi?sid=24831196
MLS002165922-01	7	11	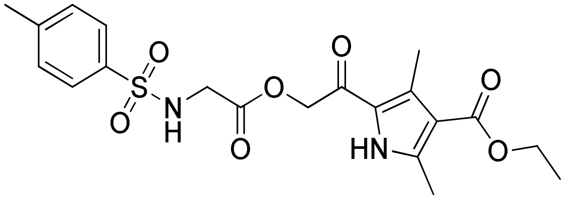	31.6228	http://pubchem.ncbi.nlm.nih.gov/summary/summary.cgi?sid=57264253
MLS000394103-01	7	11	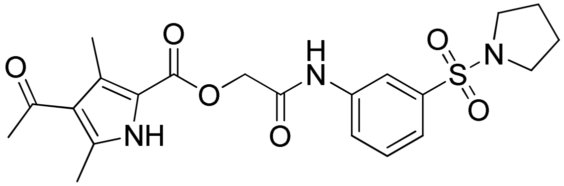	3.5481	http://pubchem.ncbi.nlm.nih.gov/summary/summary.cgi?sid=22410645
MLS000566302-01	7	11	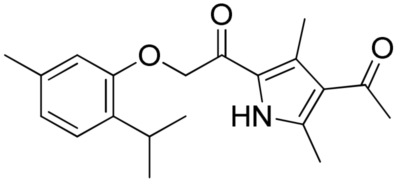	4.4668	http://pubchem.ncbi.nlm.nih.gov/summary/summary.cgi?sid=14746031

MLS000520313-01	7	11	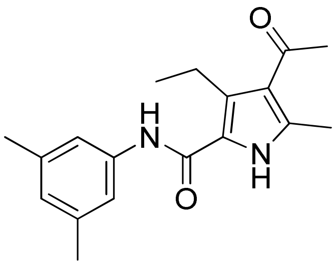	2.2387	http://pubchem.ncbi.nlm.nih.gov/summary/summary.cgi?sid=14737284
MLS000391301-01	7	11	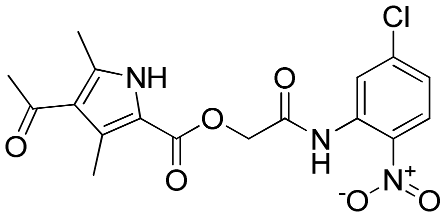	7.9433	http://pubchem.ncbi.nlm.nih.gov/summary/summary.cgi?sid=17517128
MLS002170129-01	7	11	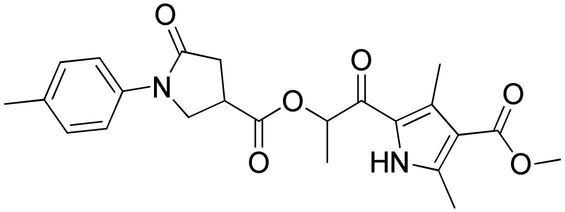	12.5893	http://pubchem.ncbi.nlm.nih.gov/summary/summary.cgi?sid=57261262
MLS002164383-01	7	11	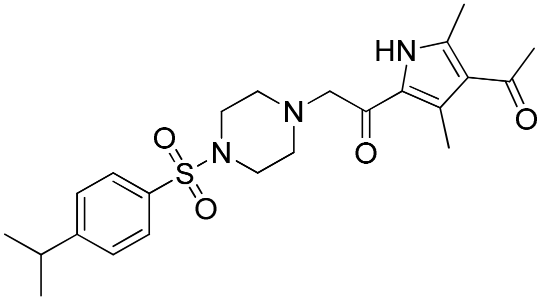	17.7828	http://pubchem.ncbi.nlm.nih.gov/summary/summary.cgi?sid=57255995

MLS000773345-01	7	11	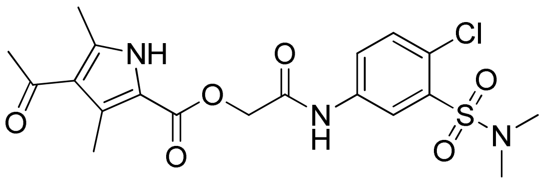	22.3872	http://pubchem.ncbi.nlm.nih.gov/summary/summary.cgi?sid=26670214
MLS000566135-01	7	11	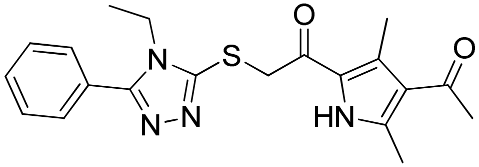	31.6228	http://pubchem.ncbi.nlm.nih.gov/summary/summary.cgi?sid=14744792
MLS002164397-01	7	11	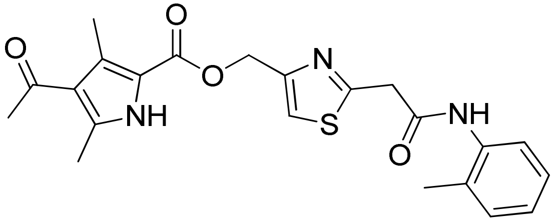	39.8107	http://pubchem.ncbi.nlm.nih.gov/summary/summary.cgi?sid=85270680
MLS000097716-01	7	11	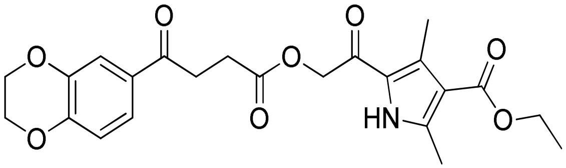	5.6234	http://pubchem.ncbi.nlm.nih.gov/summary/summary.cgi?sid=3715768
